# Influence of Electroconvection on Chronopotentiograms of an Anion-Exchange Membrane in Solutions of Weak Polybasic Acid Salts

**DOI:** 10.3390/ijms222413518

**Published:** 2021-12-16

**Authors:** Natalia Pismenskaya, Olesya Rybalkina, Ilya Moroz, Semen Mareev, Victor Nikonenko

**Affiliations:** Physical Chemistry Department, Kuban State University, 149 Stavropolskaya st., 350040 Krasnodar, Russia; n_pismen@mail.ru (N.P.); olesia93rus@mail.ru (O.R.); ilya_moroz@mail.ru (I.M.); mareev-semyon@bk.ru (S.M.)

**Keywords:** electroconvection, visualization, phosphoric acid, tartaric acid, citric acid, anion-exchange membrane, chronopotentiometric curve, current–voltage curve

## Abstract

Visualization of electroconvective (EC) vortices at the undulated surface of an AMX anion-exchange membrane (Astom, Osaka, Japan) was carried out in parallel with the measurement of chronopotentiograms. Weak polybasic acid salts, including 0.02 M solutions of tartaric (NaHT), phosphoric (NaH_2_PO_4_), and citric (NaH_2_Cit) acids salts, and NaCl were investigated. It was shown that, for a given current density normalized to the theoretical limiting current calculated by the Leveque equation (*i*/*i*_lim_*^theor^*), EC vortex zone thickness, *d*_EC_, decreases in the order NaCl > NaHT > NaH_2_PO_4_ > NaH_2_Cit. This order is inverse to the increase in the intensity of proton generation in the membrane systems under study. The higher the intensity of proton generation, the lower the electroconvection. This is due to the fact that protons released into the depleted solution reduce the space charge density, which is the driver of EC. In all studied systems, a region in chronopotentiograms between the rapid growth of the potential drop and the attainment of its stationary values corresponds to the appearance of EC vortex clusters. The amplitude of the potential drop oscillations in the chronopotentiograms is proportional to the size of the observed vortex clusters.

## 1. Introduction

Electrodialysis, electrophoresis, and capacitive deionization with ion-exchange membranes are increasingly used in hybrid membrane technologies for the separation of anions from polybasic weak acids [1,2,3,4,5,6], amino acids [7], proteins [8,9], and natural dyes [10,11], as well as from biomass, communal and livestock effluents, and from food industry waste. These methods are attractive due to the possibility of isolating these substances from strong electrolytes [12,13,14], concentration [15], fractionation [16], and the conversion from ionic into molecular forms and vice versa [5,17,18] without the use of additional chemical reagents. This is due to the ability of the listed substances (ampholytes) to enter the protonation–deprotonation reactions and to change their electric charge depending on pH. On the other hand, this same ability greatly complicates the mechanisms of ampholyte transport in systems with ion-exchange membranes, particularly of weak polybasic acid species, as compared to strong electrolytes such as NaCl. Indeed, several types of such species (molecular form, singly and doubly charged anions, etc.) can be simultaneously present in a solution. Moreover, their ratio in the bulk solution, diffusion layers, and the membrane may differ [19,20]. The factors influencing these differences have recently been intensively studied [14,21,22] in order to increase the current efficiency and find the optimal current modes, as well as the most suitable ion-exchange membranes. 

Voltammetry [12,13,20,23,24] and chronopotentiometry [12,17,19,25,26,27] seem to be the most informative and efficient methods for studying the influence of factors, such as electrolyte nature, on the behavior of systems with ion-exchange membranes in an electric field. The possibilities and benefits of these methods are comprehensively presented in a recent review by Barros and colleagues [28]. However, it should be noted that the current–voltage curves (CVC) and chronopotentiometric curves (ChP) obtained in ion-exchange membrane (IEM) systems with ampholyte solutions often differ from those obtained in IEM systems with strong electrolyte solutions [29]. CVCs have several regions of an inclined plateau [18,19,20,21]; ChPs show two or more regions of the transition time [12,14], etc. 

The mechanisms of mass transfer phenomena that predetermine the unusual shape of CVCs and ChPs of membranes in ampholyte-containing solutions, as well as the unusual (compared to strong electrolytes) behavior of membrane systems in electrodialysis processes, have recently been actively discussed in papers [20,21,23,30,31,32,33]. For example, Gally and colleagues [26] report the presence of not one, but two transition times in ChPs of anion-exchange membranes recorded in solutions containing a mixture of singly and doubly charged phosphoric acid anions. They hypothesize that the reason for the appearance of two inflection points on the ChP is the depletion of DBL, first in H_2_PO_4_^−^_,_ and then in HPO_4_^2−^ ions. Similar ChPs were obtained in a solution of Cr_2_SO_4_ [34] or copper chelates [35] at pH, which predetermines the presence of a mixture of singly and doubly charged counterions in a depleted DBL. Other discussed phenomena are: (1) more intense generation of protons and hydroxyl ions [27,36,37,38]; (2) specific interactions of carboxyl or phosphonate groups of ampholytes with fixed groups of anion-exchange membranes [26,27]; and (3) steric hindrances during the transport of large, highly hydrated polybasic acid anions in the IEM [26,27]. 

Electroconvection (EC) is another phenomenon that is often mentioned for the interpretation of CVCs and ChPs, or when discussing the regularities of the transport of ampholytes in overlimiting current modes. The EC term denotes vortex motion of a fluid in a depleted solution at the surface of an ion-exchange membrane. The occurrence of such vortices is induced by the action of the tangential component of the electric force on the electric double layer or space charge region [39]. The regularities of EC development in systems with ion-exchange membranes have been studied in sufficient detail and summarized in reviews [40,41,42]. In particular, it is known that EC develops according to the mechanism of electroosmosis of the first kind (EO I) in a non-threshold regime (including underlimiting currents) near a rough (undulated) and/or electrically inhomogeneous surface [43,44,45]. EO I is characterized by a relatively small size of electroconvective vortices. The contribution of EO I to the increase in mass transfer is usually not significant, but this phenomenon can increase the limiting current determined using the CVC. Much larger EC vortices and vortex clusters (a sufficiently complicated system of twin counter-rotating vortices which move like a single whole [46,47]) are formed by the mechanism of electroosmosis of the second kind (EO II) as a result of the interaction of the tangential component of the electric field and the extended space electric charge. The extended space electric charge is formed at the depleted solution/IEM interface under the action of the same external electric field. The above two kinds of EC are sometimes referred to as Dukhin’s and Rubinstein’s electrokinetic modes, respectively [48].

Interest in EC is quite justified because this phenomenon can provide a significant increase in the mass transfer in overlimiting current modes [40], making it possible to propose new devices for the desalination of multicomponent and dilute solutions [42,49], as well as preventing or strongly reducing scaling in the electrodialysis demineralization of multicomponent liquid media in the food industry [41,50]. Evidence of EC development in the presence of phosphoric acid anions, EDTA, heavy metal hydroxides, citrates, tartrates, and other ampholytes is revealed in investigations carried out by the groups of Pérez-Herranz and Bernardes [12,23,25,34,51], as well as Bazinet [32,50,51,52]. 

At the same time, the theory of electroconvection was developed only for membrane systems containing strong electrolytes, such as NaCl. Experimental data on the development of EC in ampholyte-containing membrane systems are fragmentary. Electroconvection, with rare exceptions [38], is identified by indirect signs, such as oscillations in the potential drop on the CVCs or ChPs. The development of EC that depends on the ability of polybasic acids to enhance the generation of protons in membrane systems has not yet been discussed in the scientific literature.

This article focuses on the study of the relationship between the intensity of EC and the rate of proton generation at the interface of the anion-exchange membrane and the solution containing anions of phosphoric, tartaric, or citric acids. 

The visualization of vortex EC clusters will be carried out in parallel with the recording of chronopotentiometric curves. The regularities of the development of electroconvection will be determined depending on the nature of the ampholyte, current density, and time elapsed since the moment the current was switched on.

## 2. Experimental

### 2.1. Membranes and Solutions

The anion-exchange membrane Neosepta AMX (Astom, Osaka, Japan) was chosen for this study because it has an undulated surface [53], which promotes the EC development [40]. This AMX membrane contains quaternary ammonium bases and small amounts of weakly basic primary, secondary, and tertiary amines as fixed groups. The entire AMX surface is available for ion transport. The heterogeneous cation-exchange membrane MK-40 (Shchekinoazot, Pervomayskiy, Russia), with fixed sulfonate groups, was an auxiliary one. The characteristics of the volume and surface of these membranes are described elsewhere and are previously summarized [54]. Some of them, including undulation parameters, are presented in the Supplementary Materials (Supplementary Materials Section S1 further in the text).

The membranes were subjected to salt pretreatment in NaCl solutions [55], then the AMX membrane was divided into 4 samples. Each of the samples was equilibrated with one of 0.02 M solutions of sodium salts of orthophosphoric (H_3_PO_4_), tartaric (H_2_T), or citric (H_3_Cit) acids, or with a sodium chloride (NaCl) solution. The pH values of the solutions ensured the maximum possible concentration of singly charged anions among other species of weak polybasic acids (Table 1). The molar fraction of the polybasic acid species at given pH values was calculated using the 1st, 2nd, and the 3rd equilibrium dissociation constants of the corresponding acid [56]. Details of the calculation, as well as the dependence of the ratio of molar fractions upon pH values, are presented in Supplementary Materials, Section S2. The designations of the solution under study correspond to the dominant fraction of the electrolyte in it.

Analytical grade crystalline salts (OJSC Vekton, St. Petersburg, Russia) and distilled water with an electrical resistance of 1.0 ± 0.1 µS cm^−1^ and pH 5.6 were used to prepare the NaCl, NaH_2_PO_4,_ and NaHT solutions. The NaH_2_Cit solution was prepared using crystalline citric acid and 0.10 M NaOH solution (OJSC Vekton, St. Petersburg, Russia).

### 2.2. Methods

#### 2.2.1. Experimental Set-Up and Processing of CVCs and ChPs

**Figure 1:**
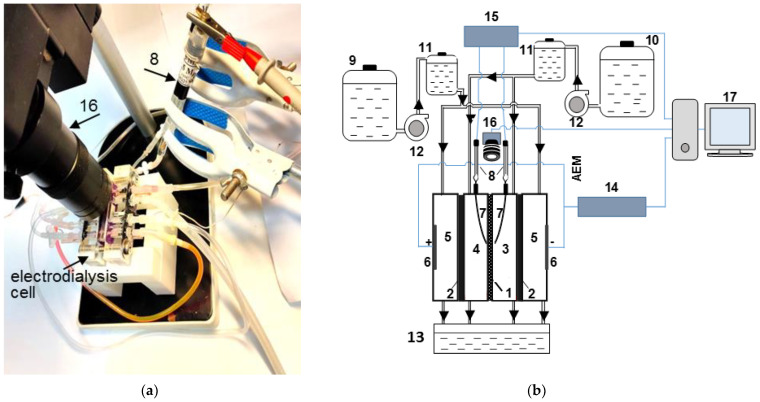
Photo (**a**) and scheme (**b**) of the experimental set-up for recording current–voltage or chronopotentiometric curves of the studied membrane in parallel with the visualization of the concentration polarization phenomena near its surface. The electrodialysis cell (top view) contains the anion-exchange membrane under study (1) and auxiliary cation-exchange membranes (2), which form the concentration, CC (3), and desalination, DC (4), compartments; platinum polarizing electrodes (6) bound the electrode compartments (5); Luggin capillaries (7) are connected to microtanks, in which measuring Ag/AgCl electrodes (8) are placed; the Dixion Instilar 1428 syringe pump (12) supplies a solution of the electrolyte under study to the electrode, concentration, and desalination compartments from the tanks (9) and (10); buffer tanks (11) prevent the pulsation of the solution, which can be caused by the use of the pump; solutions that have passed through the electrodialysis cell flow into the tank (13); the current source Keithley source meter 2400 (14) sets the current density in the electrodialysis cell: the Keithley multimeter 2010 (15) measures the potential drop between the Luggin capillaries; a CMOS camera SOPTOP CX40M optical microscope (16) equipped with a fluorescent attachment and a 180× magnifying lens registers EC vortex clusters; a computer (17) provides a digital recording of the measured characteristics.

An experimental set-up (Figure 1) and a technique similar to that described previously [57] were used to obtain CVCs and ChPs of the studied AMX membrane in parallel with the visualization of the phenomena caused by concentration polarization. This membrane forms the desalination channel, DC (4), and concentration channel, CC (3), together with the MK-40 auxiliary membranes. The membranes in the cell were arranged vertically. The solution flowed perpendicular to the direction of Earth’s gravitational field. Preliminary experiments have shown that any fluctuations of the hydraulic flow are absent in the registration zone of electroconvective vortices if the density of the electric current in the studied membrane system was zero. Detailed information on the organization of hydraulic flow is given in the Section S4.2, Supplementary Materials. The main characteristics of the four-compartment electrodialysis flow cell are presented in Table 2.

Rhodamine 6G (RG6), which fluoresces in the 540–630 nm wavelength range, was added to solutions pumped through the desalination and concentration compartments. The concentration of RG6 in these solutions was 10 μM; its particle diameter is 16 Å. At the pH of the studied solutions (Table 1), R6G dissociates, forming the Cl^−^ anion and the R6G^+^ cation [58]. The solution enriched with R6G^+^ cation (and with Na^+^ cations) has a light gray color in video frames. The solution depleted in these cations and Cl^−^ anions turns black. The resolution of the digital optical system allows one to register objects with a diameter of 20 microns or more.

The current sweep rate was 0.02 mA/cm^2^ to obtain galvanodynamic CVCs. The duration of a direct current pulse (60 s) during ChP recording was selected in preliminary experiments in order to minimize the possible adsorption of RG6 on the AMX surface. The applied current density increased from lower to higher values. The duration of the relaxation stage between current pulses was 20 min. A feed solution was pumped through the CC and DC of the electrodialysis cell during this time.

The ohmic component of the potential drop (PD) was subtracted from the obtained CVCs and ChPs in order to make a more pronounced phenomena caused by the concentration polarization of the studied membrane system. The procedure for determining this reduced PD is described in Supplementary Materials, Equations (S73) and (S74).

#### 2.2.2. Limiting Current and Diffusion Boundary Layer Calculation

For NaCl and NaH_2_PO_4_ solutions, which contain 100% and 98% of singly charged anions (Table 1), respectively, the limiting current, *i*_lim_*^theor^*, and the diffusion boundary layer (DBL) thickness, *δ^theor^*, were calculated using the Leveque equation obtained within the framework of the convective–diffusion model [59]:

(1) $$i_{\lim}^{theor}=\frac{z_{1}FDc_{1}^{0}}{h(T_{1}-t_{1})}\left[1.47{\left(\frac{h^{2}V_{0}}{LD}\right)}^{1/3}\right]$$

(2) $$\delta^{theor}=0.68h{\left(\frac{LD}{h^{2}V_{0}}\right)}^{1/3}$$

Here *F* is the Faraday constant, *D* is the diffusion coefficient of electrolyte, *z*_1_ and *t*_1_ are the electric charge and electromigration counterion transport number in a solution at infinite dilution, respectively, $c_{1}^{0}$ is the molar concentration of counterion in bulk solution, *V*_0_ is the mean linear solution flow velocity in DC, *h* is the intermembrane distance, and *L* is the polarized (by electric current) DC path length. The counterion transport number in the membrane, *T*_1_, was considered to be equal to 1.

In the case of NaHT and NaH_2_Cit solutions, the equation for the ternary electrolyte was used [60]:

(3) $$i_{\lim}^{theor}=\frac{F}{\delta }\sum_{k=1}^{2}\left(1-\frac{z_{k}}{z_{A}}\right)D_{k}z_{k}c_{k}^{0}$$

where *D_k_*, *z_k_*_,_ and $c_{k}^{0}$ are the diffusion coefficient, charge, and molar concentration of counterion *k*, respectively (*k* = 1, 2); *z_A_* is the charge number of the coion. An equation similar to Equation (2) used to determine *δ^theor^*. The difference compared to NaCl and NaH_2_PO_4_ solutions was in the use of the diffusion coefficient of the ternary electrolyte, *D_ter_*:

(4) $$D_{ter}=\left[\left(1+\left|\frac{z_{1}}{z_{A}}\right|\right)D_{1}N_{1}+\left(1+\left|\frac{z_{2}}{z_{A}}\right|\right)D_{2}N_{2}\right]\cdot t_{A}$$

where $N_{i}=z_{i}c_{i}^{0}/z_{A}c_{A}^{0}$ is the equivalent fraction of counterion *i* in the bulk solution. The derivation of Equations (3) and (4), as well as the values of *D*_1_, *D*_2_, *D_ter_*_,_ and *t_A_* that were used for the calculations are presented in Table S3, Supplementary Materials. Similar approaches for determining the limiting currents were applied by Gally and colleagues as well as Chandra and colleagues, who studied the transport of phosphoric [26] or citric [14] acid anions in systems with AEM. 

Table 3 summarizes the calculated values of these limiting currents and DBL thicknesses, as well as the limiting currents (*i*_lim_*^exp^*) found using CVCs (the procedure for determining *i*_lim_*^exp^* is explained in Section 3.1).

The limiting currents calculated using Equations (1) and (2) or Equations (2) and (3) have the same physical basis. These limiting currents correspond to the state of a membrane system, in which the salt concentration at the IEM surface becomes negligible compared to the bulk concentration. The electric charge is carried by electromigration, diffusion, and convection of the ions present in the bulk solution. Any chemical reactions are not taken into account. 

#### 2.2.3. Determination of EC Vortex Zone Thickness

**Figure 2:**
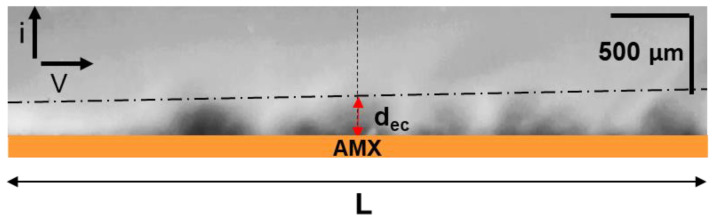
An example of the average EC vortex zone thickness, d_ec_, determination from the video frame. The vertical dotted line shows the middle of the polarizable surface of the AMX membrane facing the desalination compartment. The dash-dotted line is drawn along the tops of the vortex clusters.

The EC vortex zone thickness, d_ec_, was determined from video frames as shown in Figure 2. A similar method has been applied previously [61].

#### 2.2.4. Conductivity Measurements

The conductivity, ᴂ, and electrical resistance, *R*, of the AMX membrane was determined by the differential method using a clip-cell [55] in 0.02 eq dm^−3^ (*C* = *C* = *z_i_* $c_{1}^{0}$) solutions of the studied electrolytes. This concentration was chosen to compare the conductivity of the membrane at a constant number of electric charge carriers in the external solution. The measurements were carried out in the solutions under study with pH 3.7 ± 0.1 (NaHT), 4.6 ± 0.1 (NaH_2_PO_4_), and 4.0 ± 0.1 (NaH_2_Cit). In addition, solutions with pH 7.0 ± 0.1 (NaHT), 9.5 ± 0.1 (NaH_2_PO_4_), and 9.0 ± 0.1 (NaH_2_Cit) were used. In this case, doubly charged anions of tartaric or phosphoric acid, or triply charged anions of citric acid, were predominantly in the solution and in the membrane. The ohmic PDs of the membrane were estimated from these values of electrical resistance (Δφ_Ω_ = *R* × *I*) at a given current, *I*. Some details of measurements are given in Section S4.1, Supplementary Materials.

## 3. Results and Discussion

### 3.1. Background: Proton Generation in AEM/Ampholyte Solution Systems

**Figure 3:**
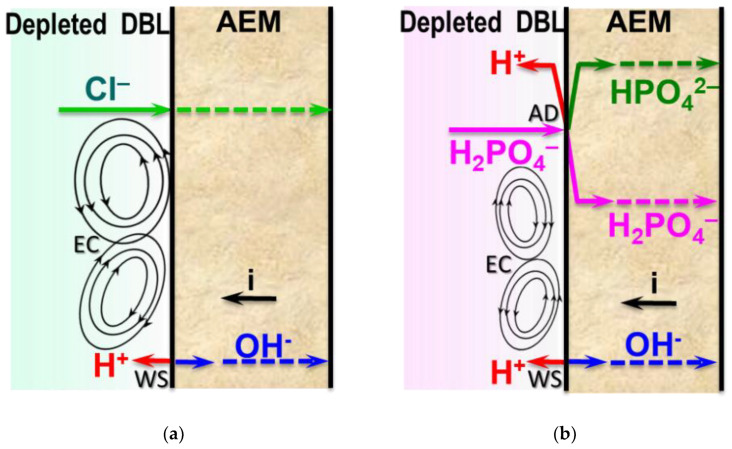
Schematic representation of the phenomena developing at the anion-exchange membrane/depleted solution interface in overlimiting current modes for the cases of a strong electrolyte solution (**a**) and a solution containing singly charged anions of a polybasic weak acid (**b**). WS is a water splitting mechanism and AD is an “acid dissociation” mechanism of proton generation; EC is electroconvection.

Two mechanisms for the generation of protons and hydroxyl ions are possible in membrane systems containing species of weak polybasic acids [12,23,26]. The first does not differ from water splitting (WS) [36,41,62,63,64], which is well known for the case of strong electrolytes (NaCl, KNO_3_, etc.). The generation of H^+^ and OH^−^ ions occurs in the threshold mode (at currents close to the limiting one and higher) and is carried out with the catalytic participation of membrane fixed groups (Figure 3a). The second mechanism is characteristic only for membrane systems that contain substances participating in protonation–deprotonation reactions (ampholytes) [24,65,66]. Upon entering the AEM, singly charged anions of polybasic weak acids (which are ampholytes) are deprotonated with the formation of protons and doubly charged anions. The protons are excluded from the membrane due to the Donnan effect (Figure 3b). Multiply charged anions are transported through the AEM, increasing the recorded current density. However, this increase has almost no effect on the target component transport, such as elemental phosphorus in the case of NaH_2_PO_4_ [24], because the number of anions involved in the transfer does not change. This mechanism, which for brevity can be denoted as “acid dissociation” (AD), takes place under any current mode. Enhancing the proton current is controlled by the reaction rate constants, which limit the dissociation of the acid at stages 1, 2, and 3 [67], and the ion-exchange capacity of the membrane, IEC. The ion-exchange capacity controls the Donnan exclusion of coions (protons) [68] and limits the number of multiply charged anions that can participate in the transfer of electric charge in the membrane. The smaller difference between equilibrium constants, pK_ai_, for the first and second acid dissociation steps, the more doubly charged anions are present in the membrane [19] for a given *i*/*i*_li_*^rLev^* ratio. For example, even in the absence of an applied electric field, the molar fraction of doubly charged anions in AMX is 38.2% (NaH_2_PO_4_ solution, pH 4.6) and 92.9% (NaHT solution, pH 3.7). In the case of a NaH_2_Cit solution with pH 4.0, the membrane contains 70.6% of doubly charged anions and 26.2% of triply charged anions. The fraction of multiply charged ions in the membrane increases with an increase in the current density. These estimations take into account ion-exchange equilibria and pK_ai_ values for each acid under study. For more information, see Section S3.1.2, Supplementary Materials.

Our experiments (see Section S4.1, Supplementary Materials) show that for a given *i/i*_lim_*^theor^* ratio, the proton transport numbers in the solution adjacent to the AMX increase in the sequence: NaCl < NaHT < NaH_2_PO_4_ < NaH_2_Cit (Table 4).

The minimum values of the proton transport numbers in the near-membrane NaCl solution are explained by the fact that only water splitting is a source of protons. Both the WS and AD mechanisms can occur simultaneously [67] in overlimiting current modes in the case of polybasic acid salt solutions (Figure 3b).

### 3.2. Current–Voltage Curves 

CVCs of the AMX membrane obtained in 0.02 M solutions of the studied electrolytes are shown in Figure 3. At least two inclined plateaus, II_1_ and II_2_, are observed on these curves.

**Figure 4:**
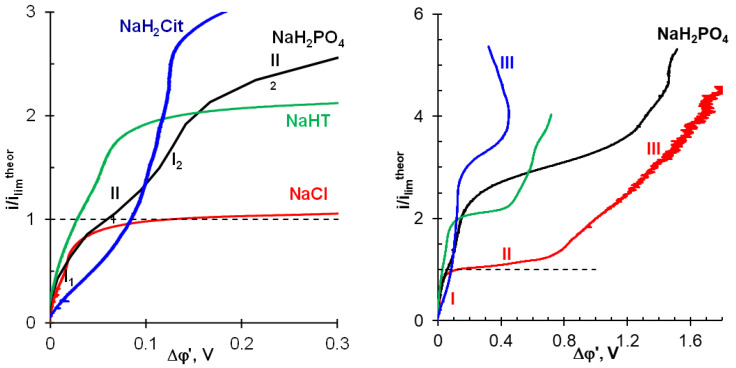
Current–voltage curves of the AMX membrane in 0.02 M solutions of the studied electrolytes. The applied currents are normalized to the limiting current *i*_lim_*^theor^* (Table 3), calculated using Equations (1) and (2) or (2)–(4). The dotted line shows the calculated limiting current. The intersection of the tangents to the initial region I and the region II of the inclined plateau gives the value of an experimental limiting current, *i*_lim_*^exp^*, in the case of NaCl solution. The intersection of tangents to the regions I_1_, II_1_ or I_2_, II_2_ gives the value of *i*_lim_*^exp^* in the case of NaHT, NaH_2_PO_4_, and NaH_2_Cit solutions.

The first limiting current, *i*_lim 1_*^exp^* (found from the intersection of the tangents to the initial region I_1_ and the region of the inclined plateau II_1_ and close to *i*_lim_*^theor^*, Table 3) has a nature similar to the membrane system with a strong electrolyte, such as NaCl. *i*_lim 1_*^exp^* is caused by electrolyte depletion in the near-membrane solution at the AEM/depleted DBL interface, as well as the saturation of the partial current of singly charged polybasic acid anions in the membrane [24,66]. The results of mathematical modeling of the total and partial CVCs, which confirm this conclusion, are presented in Figure S6, Supplementary Materials. The more protons that are released into the depleted solution, the “less pronounced” the inclined plateau II_1_ on the CVC is (Figure 4). A further increase in the current in region I_2_ takes place due to an increase in the electric charge of anions in the membrane, as well as the transfer of the electric charge at the AEM/depleted DBL interface by protons coming from the AEM.

The second limiting current, *i*_lim 2_*^exp^*, in the cases of NaHT and NaH_2_PO_4_, is identified by a well-pronounced inclined plateau, II_2_, in the vicinity of 2*i*_lim_*^theor^*. It corresponds to a state in which the AEM is almost completely saturated with doubly charged anions; the maximum rate of proton generation is achieved by the AD mechanism, and there are no other sources of increasing the electric charge carried by ions. According to estimates made previously [67], in the case of a NaH_2_PO_4_ solution, the kinetic rate of proton generation by the AD mechanism is limited due to a low rate constant of phosphoric acid dissociation in the 3rd step (Table S2 in Supplementary Materials); tartaric acid does not have the 3rd dissociation step. An increase in *i*_lim 1_*^exp^* and *i*_lim 2_*^exp^* in comparison with theoretical values (Table 3) is caused by the exaltation of the limiting current [66]. This phenomenon is caused by the creation of an additional electric field with protons excluded from AEM. The field attracts additional amounts of salt anions to the AEM/diluted DBL interface [69].

In the case of a membrane system with NaH_2_Cit solution at *i* < *i*_lim_*^theor^*, the reason for the higher resistance (the higher PD) compared to other studied systems is apparently the more intense accumulation of the molecular form of citric acid in diluted DBL due to the reaction: H_2_Cit^−^ + H^+^ → H_3_Cit. This accumulation is caused by the simultaneous presence in the membrane of both singly and doubly charged anions, the deprotonation of which is characterized by very high kinetic constant rates of 2·10^5^ s^−1^ and 4·10^3^ s^−1^, respectively (Table S2 in Supplementary Materials). Moreover, the transformation of HCit^2−^ anions into Cit^3−^ does not meet kinetic limitations [67]. Therefore, in the case of the NaH_2_Cit solution, an inclined plateau in the CVC is observed only in the vicinity of 3*i*_lim_*^theor^*, where, apparently, the maximum flux of H^+^ ions generated by the reaction HCit^2−^ → Cit^3−^+ H^+^ is reached. 

The “length” of the inclined plateau is determined by the PD in the point of intersection of the tangents to the region of the inclined plateau II and the overlimiting region III in the case of strong electrolytes (Figure 4). It should be noted that, as a rule, this “length” is considered as the threshold value necessary for the occurrence of non-equilibrium EC, developed by the mechanism of electroosmosis of the second kind [40,44,70,71,72]. As mentioned in the Introduction, this type of EC is characterized by the formation of large vortices or vortex clusters. They mix the depleted solution, causing a decrease in the concentration polarization and an increase in the conductivity of region III as compared to region II (Figure 4) in the case of NaCl. On the CVC region III, PD oscillations, the amplitude of which increases with increasing current density, are attributed to the emergence and gradual enlargement of EC vortex clusters.

The concept of the dominant effect of EC on the behavior of membrane systems in overlimiting current modes, as a rule, is extended to solutions containing polybasic organic and inorganic acids or other ampholytes [14,25,26,32,34,41,73]. At the same time, the higher conductivity of the AMX/NaH_2_Cit solution system observed in region III of the CVC in comparison to region I suggests another mechanism for the increase in the conductivity of depleted DBL in overlimiting current modes. 

### 3.3. Chronopotentiograms

**Figure 5:**
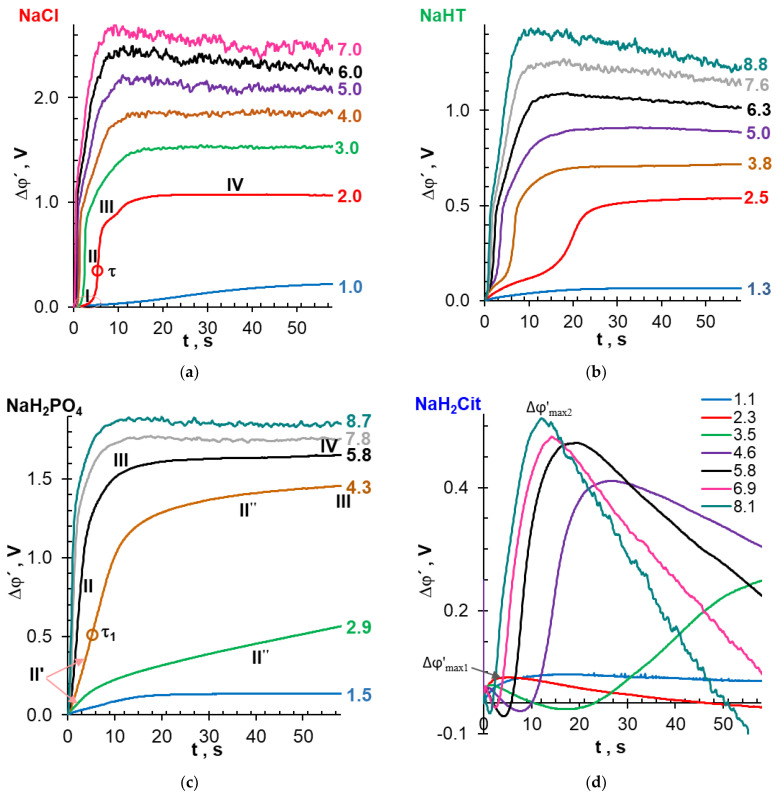
Chronopotentiograms of the AMX membrane in 0.02 M solutions of NaCl (**a**), NaH_2_PO_4_ (**b**), NaHT (**c**), and NaH_2_Cit (**d**). The numbers indicate the *i/i*_lim_*^theor^* values at which the corresponding curves were obtained.

Chronopotentiograms (ChPs) (Figure 5) make it possible to trace the development of concentration polarization phenomena after switching on a direct current pulse. The main achievements in the field of ChP interpretation are summarized in a recent review [28]. With regard to solutions of strong electrolytes (NaCl), ChPs presented in Figure 5a are interpreted as follows. A sharp (within a few hundredths of a second) increase in PD, which is observed immediately after switching on the current, is determined by the electrical resistance of the AEM and the adjacent non-polarized layers of the solution, located between the Luggin capillaries. This ohmic region was subtracted from all analyzed ChPs. The next region of decelerated increase in PD (indicated by the index I) characterizes the formation of electrolyte concentration profiles in depleted and enriched DBLs, adjacent to the membrane surfaces. The local maximum in the beginning of this region (see the inset in Figure 5a) can be attributed to the emergence of EC, which develops by the EO I mechanism. Similar maximums were observed previously [74]. Region II on ChPs appear in overlimiting current modes and corresponds to a rapid increase in PD due to the depletion of electrolyte solution near the membrane surface. The inflection point in region II (up to several tens of seconds) corresponds to the so-called transition time, τ. WS, EC, and other phenomena induced by the concentration polarization affect potential drop at the time, t, higher than the transition time (t > τ). The growth of the PD is slowed down (region III) due to the appearance of new electric charge carriers (H^+^ or OH^−^ ion) or the electroconvective delivery of a more concentrated solution from the bulk of the depleted DBL to the membrane/solution interface. The development of these phenomena leads to the achievement of stationary values of PD (region IV). PD oscillations in region IV are attributed to the emergence of large EC vortex clusters.

Chronopotentiograms of AMX, obtained in solutions containing species of weak po-lybasic acids (Figure 5b–d) differ from curves recorded in the NaCl solution. In our experiments, region I loses its local maximum in the NaHT solution (Figure 5c) and becomes more diffuse in the NaH_2_PO_4_ solution (Figure 5b) compared to NaCl (Figure 5a). “Blurring” of region I apparently increases with an increase in the proton concentration (generated by the AD mechanism [67]) in the depleted DBL (Table 4). Otherwise, the shape of ChPs obtained in NaHT and NaH_2_PO_4_ solutions differs little from the shape of curves obtained in moderately dilute solutions of strong electrolytes [27,29,75]. Only two features are exceptions: (1) at current densities 2 < *i*/*i*_lim_*^theor^* < 5, an inclined region II′′ appears on ChPs obtained in a NaH_2_PO_4_ solution; (2) the amplitude of PD oscillations in the case of NaHT and NaH_2_PO_4_ solutions is less than in the AMX/NaCl solution system for given values of *i*/*i*_lim_*^theor^*. 

A comparison of the double-pulse chronopotentiograms of the AEM/ NaH_2_PO_4_ solution system [76] with the results of calculations of concentration profiles in the membrane and adjacent DBLs using a stationary model [20] (see Figure S7 in Supplementary Materials) provides the basis for the following hypothesis. The region *II″*, which has a duration that may reach several hundred seconds, characterizes the stage when the membrane is saturated with HPO_4_^2−^ anions and the molar fraction of singly charged anions becomes negligible.

The deprotonation of doubly charged HPO_4_^2−^ anions with the formation of triply charged PO_4_^3−^ anions has kinetic limitation (see the Table S2 with rate constants in Supplementary Materials). Therefore, the rate of proton generation by the AD mechanism also tends to be saturated. Protons are one of the main charge carriers in a depleted boundary solution. Thus, the rate of proton exclusion from the membrane controls the recorded potential drop. A slow rearrangement of the concentration profiles of electric charge carriers inside the membrane limits the concentration of protons at the interface of the AEM/diluted solution. The intense WS and/or EC developing at higher current densities eliminates the carrier deficit at the interface of the AEM/depleted solution. As a result, ChPs acquire a shape characteristic of strong electrolytes if *i*/*i*_lim_*^theor^* > 5 (Figure 5b). However, the development of a non-stationary mathematical model for the transport of ampholytes in systems with anion-exchange membranes is required to confirm this hypothesis.

The ChPs of the AMX/NaH_2_Cit system (Figure 5d) obtained in overlimiting current modes (*i*/*i*_lim_*^theor^* > 1) are characterized by very low PD values that are not observed in other studied systems. A decrease in PD can be caused by a high concentration of protons in the depleted DBL since the mobility of protons is an order of magnitude higher than that of citric acid anions (see Table S3 in Supplementary Materials). In addition, our measurements show that as the resistance of the membrane decreases and the electrical conductivity increases due to the enrichment of the AMX internal solution with triply charged citric acid anions (Table 5). The calculated difference of ohmic PD values in the feed NaH_2_Cit solution (pH 4.0) and in the solution Na_x_H_(3−X)_Cit with pH 9.0, providing the presence of mainly triply charged anions in AMX, is equal to 349 mV. This value is comparable to the Δφ′_max2_, which is equal to 410 mV at *i* = 4.6*i*_lim_*^theor^* (Figure 5d). At the same time, the difference, caused by the conversion of all species of phosphoric and tartaric acid into doubly charged anions, is about 20 mV against 1450 mV (AMX/NaH_2_PO_4_ system, Figure 5c) and 716 mV (AMX/NaHT system, Figure 5b), respectively. Thus, an increase in the electric charge of the counterion in the membrane caused by the AD mechanism has an insignificant effect on the stationary PD values in the AMX/NaH_2_PO_4_ and AMX/NaHT systems. These data confirm the results obtained earlier [19,76]. 

The minimums and maximums are another specific feature of ChPs in the case of the NaH_2_Cit solution. It appears that the first maximum of Δφ′_max1_ occurs due to the delay in the delivery of protons from the membrane to the depleted DBL after the current is switched on. An increase in the current density contributes to an increase in the proton transfer rate, which leads to a shift of the first maximum to lesser times (Figure 5d). The replacement of less mobile citric acid anions in dilute DBLs with more mobile protons causes a decrease in the registered PD as compared to the ohmic Δφ_Ω_. As a result, the reduced ChP (from which the ohmic component has been subtracted) acquires negative PDs values. The subsequent increase in the PD is caused by the saturation of the membrane with triply charged anions, Cit^3−^, which results in an increasing deficit of charge carriers (protons) at the AEM/depleted solution interface.

The second maximum, Δφ′_max2_, and the subsequent decrease in PD are observed on the ChP (Figure 5d) at the same currents (*i/i*_lim_*^theor^* > 4), which corresponds to region III on the CVC (Figure 4), with a negative differential resistance defined as the negative derivative d(Δφ′)/d*i*). These phenomena can be caused by the development of intense WS and/or EC, which sharply reduces the already low electrical resistance of the AMX/ NaH_2_Cit system. PD oscillations in this ChP region are observed at *i* > 6.9*i*_lim_*^Lev^* that indirectly confirm the development of EC. 

To discuss the influence of EC on the shape of the ChP, let us compare the characteristic points of the chronopotentiometric curves with the corresponding video frames. These ChPs were obtained at *i/i*_lim_*^theor^* = 4.2 ± 0.4. The applied current densities correspond to the “overlimiting” regions III on the CVCs. The range of these currents is highlighted by the shaded area in Figure 3.

### 3.4. Comparative Analysis of Characteristic Points on Chronopotentiograms and Results of EC Vortex Zone Visualization

Before the analysis of video frames, recall that areas lighter than the general background correspond to increased (compared to the bulk) concentrations of cations and anions. The darker areas correspond to a solution with a low concentration of the fluorescent agent, R6G^+^. The same areas are depleted in the ions of the investigated electrolyte due to the local electroneutrality. Thus, the darker the observed color, the lower the electrolyte concentration.

**Figure 6:**
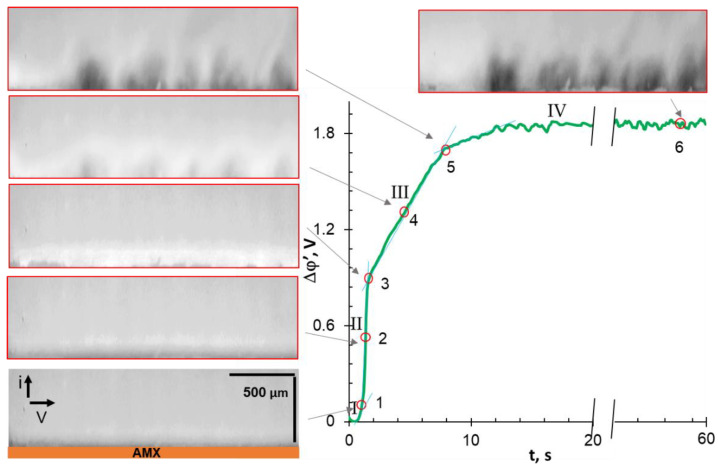
Characteristic points of the ChP and the corresponding time-lapse snapshots of the depleted solution adjoining the anion-exchange membrane. The case of the AMX membrane in a 0.02 M NaCl solution, *i/i*_lim_*^theor^ =* 4.0 (I = 13.56 mA cm^−2^).

**Figure 7:**
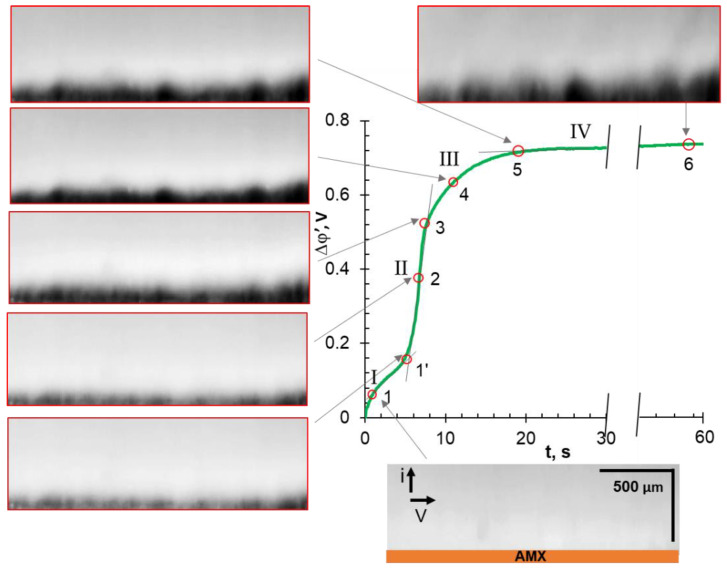
Characteristic points of the ChP and corresponding time-lapse snapshots of the depleted solution adjacent to an AEM surface. The case of an AMX membrane in a 0.02 M NaHT solution; *i/i*_lim_*^theor^ =* 3.8 (I = 7.68 mA cm^−2^).

In the cases of NaCl (Figure 6) and NaHT (Figure 7) solutions, the analyzed characteristic points on ChPs correspond to PD at the intersection of tangents to regions I and II (1); at the inflection point in region II (2); at the intersection of tangents to regions II and III (3); at the geometric center of region III (4); at the intersection of tangents to regions III and IV (5); and in region IV (6), 58 s after switching on the current. This current pulse duration is sufficient to achieve steady-state PD values in these membrane systems.

Let us first consider the video frame that corresponds to point (1) of the chronopotentiogram obtained in the AMX/NaCl system (Figure 6). A dark stripe appears in the layer adjacent to the membrane, which is an indicator of solution desalination. At point (2), corresponding to the transition time, the dark stripe reaches a maximum thickness of about 100 μm, which is consistent with the average thickness of the calculated diluted DBL (Table 3). It should be noted that the near-surface solution becomes lighter than that recorded at point (1), most likely due to the mixing of this solution with small vortices developing by the EO I mechanism [45,74,77]. It is known that such mixing leads to an increase in transition time, τ [74], which characterizes the achievement of a critically low concentration of salt counterions at the AEM surface [75]. Point (3): only small rounded blotches of dark (NaCl-depleted) areas surrounded by a lighter, unevenly colored strip are visualized near the AMX surface. The vortex motion of a fluid can be seen in the video (see Video S1 in Supplementary Materials) within this light strip. The motion indicates the formation of vortex clusters of 20 μm and more in size (the minimum size determined by the capabilities of the used equipment). The enlargement of these individual clusters between points (3) and (5) and, accordingly, the delivery of a more concentrated solution from the bulk to the membrane, leads to a slowdown in PD growth in region III of the ChP. At point (5) of the ChP, individual clusters combined into chaotic vortex structures, the sizes and appearance of which vary slightly with time in region IV, illustrated by point (6). The establishment of the constant EC vortex zone thickness and the constant rotation rate of the vortices are the reason for the attainment of stationary values of PD in the studied system. The observed development of EC vortices after switching on the current is in good qualitative agreement with both the results of visualization of concentration profile disturbances performed by the laser interferometry method [78,79], and with the results of 2D mathematical modeling [80,81,82].

It should be noted that point (2) on the ChP (Figure 6) corresponds to the beginning of WS, if the studied AEM is in the NaCl solution. This conclusion follows from the results of parallel measurements of ChPs and the pH of the solution at the inlet and outlet of the desalination channel formed by the AMX and MK-40 membranes [74] or similar membranes [76,83]. The acidification of the solution, which depends on WS intensity, increases up to point (5) on the ChP, and then reaches saturation at times corresponding to region IV. The reason for this saturation is the establishment of vortex clusters of constant size and a constant delivery rate of a sufficiently concentrated solution from the bulk to the AEM surface. It is known that WS at the AEM/depleted DBL interface may reduce electroconvection [71,84,85]. Indeed, H^+^ cations have an electric charge opposite to the space charge, which is formed at the membrane surface in overlimiting current modes, and therefore reduces its density [44,86]. At the same time, the contribution of protons to electric charge transfer in a depleted NaCl solution near the AMX surface is relatively small (Table 4). Therefore, WS does not appear to have a dramatic effect on EC intensity.

As can be seen from Figure 7, no changes in the color of the depleted NaHT solution are observed at point (1), which is absent in the ChPs obtained in other solutions. It is likely that the exclusion of protons from the membrane delays the depletion of the RG6^+^ cation in the near-surface solution. Nevertheless, the video frame corresponding to point (1′) is characterized by a thicker (as compared to NaCl solution) depleted layer, in which darker areas alternate with blurred lighter areas. Most likely, this “blurring” is caused by EO I, which occurs at a higher (about 120 mV) PD as compared to a NaCl solution (60 mV). The picture (Figure 7) observed at points (2)–(6) repeats the one already discussed for the NaCl solution. The difference lies in the darker color of the depleted layer and the larger size of the dark zones, which are characterized by a low fluid rotation rate as following from mathematical simulations in the case of the NaCl solution [80,81,82]. Noteworthy is the fact that at points (4) and (5) in the depleted zones have a “blob” shape, and at point (6), which corresponds to the stationary state, these clearly determined zones become blurred. In a recent paper [87], Stockmeier and colleagues (who performed 3D modeling and visualization of EC at the surface of a cation-exchange membrane in CuSO_4_ solution) explained this phenomenon by a change in the vortex structure from vortex rolls to vortex rings.

**Figure 8:**
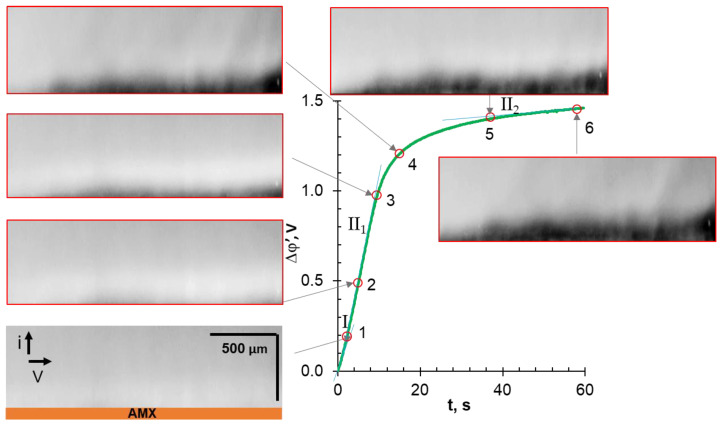
Characteristic points of ChPs and the corresponding time-lapse snapshots of the depleted solution adjacent to an AEM surface. The case of an AMX membrane in a 0.02 M NaH_2_PO_4_ solution; *i/i*_lim_*^theor^ =* 4.3 (I = 7.96 mA cm^−2^).

In a NaH_2_PO_4_ solution (Figure 8), the development of vortex structures occurs with a time delay in comparison to that observed in a NaHT solution (Figure 7). At point (4), separate zones of a highly depleted solution, which have a “blob” shape, appear only at the end of the investigated desalination channel (see Video S2 in Supplementary Materials), that is, where the concentration of the electrolyte solution is minimal. These zones expand over the entire AMX surface only at point (5), which corresponds to the IIʹ region on ChP, in contrast to the systems discussed earlier.

As mentioned in Section 3.2, this delay in the development of EC seems to be caused by the more intense generation of protons (Table 4). These protons affect the electric charges of the dense and diffuse regions of the electric double layer, worsening the conditions for the development of EO I in the first few seconds from the moment the current is switched on. Further, (at t > τ) a decrease in the space charge density worsens the conditions for the development of EO II.

**Figure 9:**
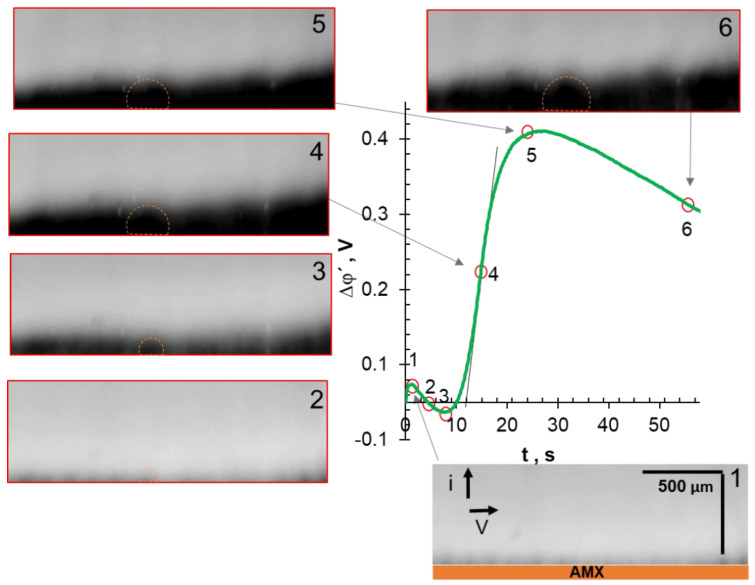
Characteristic points of ChPs and the corresponding time-lapse snapshots of the depleted solution adjacent to an AEM surface. The case of an AMX membrane in a 0.02 M NaH_2_Cit solution; *i/i*_lim_*^theor^ =* 4.6 (I = 8.00 mA cm^−2^).

The proton generation, which is most intense in the case of the AMX/NaH_2_Cit system (Table 4), has a detrimental effect on EC development (Figure 9). The extremely dark color of the depleted zones near the membrane surface, which is observed at points (3)–(6) on the ChP, indicates the weakest mixing of the solution in comparison with other studied systems. Dark, blob-shaped segments, which indicate clustering of EC vortices, appear only at points (5) and (6). Moreover, the deep black color of the near-membrane solution at point (6) allows one to conclude that the electroconvective delivery of a more concentrated solution can hardly be the reason for the decrease in PD observed in this ChP region. 

### 3.5. Influence of the Electrolyte Type on the Development of Electroconvection

We simulated the development of electroconvection at the undulated surface of an AMX anion-exchange membrane during electrodialysis desalting of a 0.01 M NaCl solution. This low concentration is used to reduce the calculation time to an acceptable level. The calculation was carried out using the 2D so-called basic model [80], which is based on the Nernst–Planck–Poisson–Navier–Stokes equations and takes into account the electroconvective transfer of ions and fluid. Calculations were performed using the commercially available COMSOL Multiphysics 5.5 software and the membrane system parameters shown in Table 2. Some details are presented in Section S3.3, Supplementary Materials.

**Figure 10:**
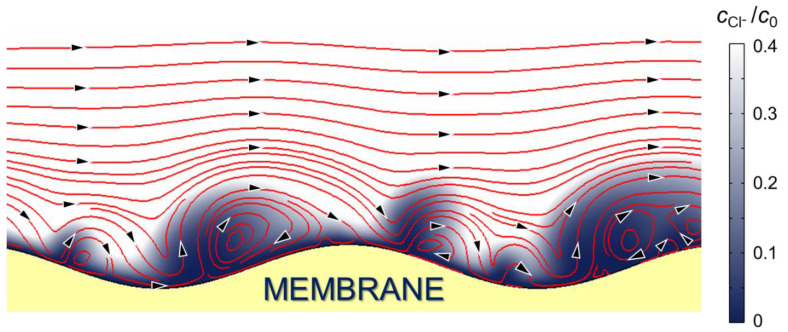
Simulated Cl^−^ ions concentration profile (shown by shades of gray) at the undulated surface of an AMX membrane in the desalination channel of an electrodialysis cell. The red lines indicate the distribution of fluid velocity streamlines; the direction of current flow is shown with black arrows. The calculation was carried out using the so-called basic model [80] for a 0.01 M NaCl solution at *i*/*i*_lim_*^theor^* = 1.7. The parameters used for the calculation are given in Table 2.

According to the results of mathematical modeling, the darkened zones of the most depleted solution (and, accordingly, the largest space charge regions) are localized at the “valleys” of the ion-exchange membrane surface. These zones are characterized by the lowest fluid rotation rate. The EC vortices, whose velocity at a sufficient distance from the membrane surface coincides with the forced flow velocity, are essentially bigger than that rotating in the opposite direction. Such a structure determines the “blob” shape of the darkened depleted zones. The forced fluid flow, which bends around the depleted zones, moves away from the membrane over the “valleys” and periodically approaches the membrane over the “hills” on its surface. Vortex clusters are destroyed periodically under the influence of the forced flow of the solution. However, after a while, they reappear practically in the same place where the depleted solution localized between the “hills” of the surface. Similar results were obtained in articles [83,84] for the geometrically inhomogeneous surface of ion-exchange membranes (Figure 10).

**Figure 11:**
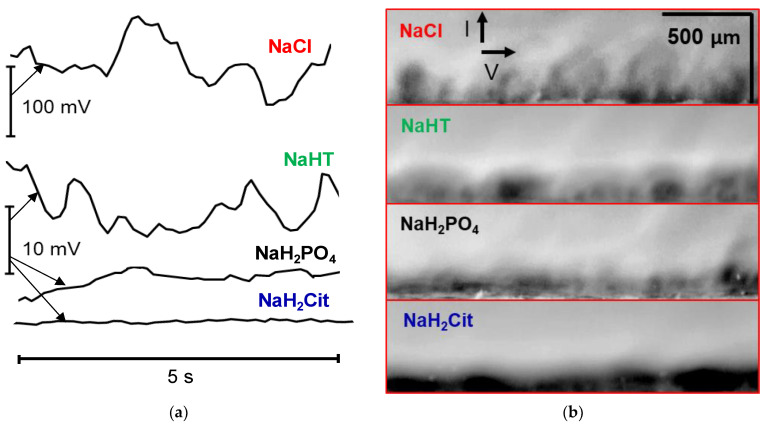
Oscillations of the PD on chronopotentiograms in a stationary state (NaCl, NaHT, and NaH_2_PO_4_ solutions) or near a stationary state (NaH_2_Cit solution) (**a**) and the time-lapse snapshots obtained at the corresponding time (**b**); *i/i*_lim_*^theor^ =* 6.0 ± 0.2.

Video frames with the visualization of EC vortices are in good agreement with the results of mathematical modeling. At the moments of the video (see Video S3 in Supplementary Materials) corresponding to the time of a stationary state (or close to it), it is clearly seen that EC vortices, that form in the darkened zones eject the depleted solution from the “valleys” into the bulk flow (Figure 11b). In the case of the NaCl solution, the result of these ejections is an almost complete periodic “blurring” of the depletion zone. A new depleted zone in the electrolyte is formed in about 0.7 s at the same place. Moreover, the shape of this zone in the near-surface solution repeats the shape of the previous (not blurred) zone. The intensity of ejections of the depleted solution into the bulk flow and the size of the “prominences” decrease, while the sizes of the dark zones, characterized by low rates of electroconvective mixing of the depleted solution, grow in the sequence NaCl > NaHT > NaH_2_PO_4_ > NaH_2_Cit. The amplitude of PD oscillations observed on the ChPs (obtained in parallel with the video) decreases (Figure 11a) in the same sequence. The dark, “blob-shape” segments indicate an area of low ion concentration in the depleted DBL. These segments, protruding against the general dark background, are clearly visible in the case of NaH2Cit. Moreover, their size is quite comparable with the size of vortex clusters in the solutions of other electrolytes.

These data allow us to assume that, in the case of an undulated membrane, the reason for the PD oscillations may be the periodic appearance and disappearance of vortex clusters at the same place of the surface directly under the Luggin capillaries. This scenario differs from that known for membranes with a smooth and electrically homogeneous surface. The results of mathematical modeling [40,80] and experiments [28,88] show that in this case, vortex clusters slip along the membrane/dilute DBL boundary due to the forced convection, causing the alternation of a more concentrated and more depleted solution under the Luggin capillaries.

**Figure 12:**
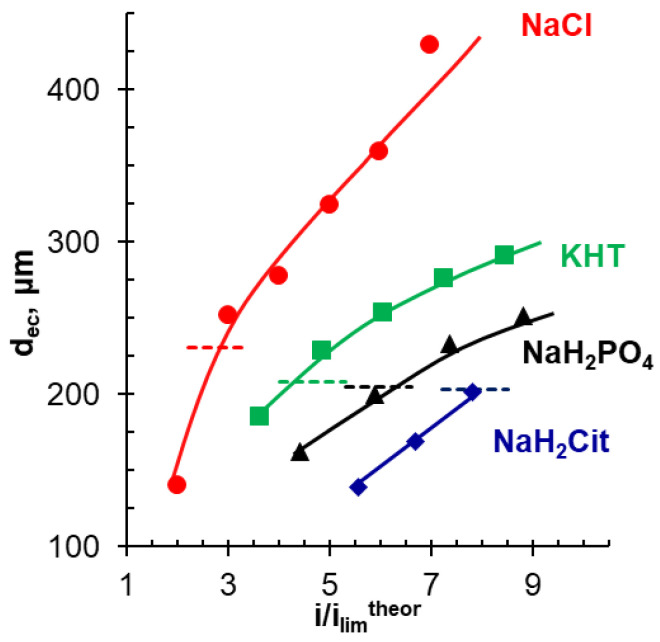
EC vortex zone thickness, d_ec_, vs current density normalized by theoretical limiting current, calculated using Equations (1) and (3). The dashed lines represent the depleted DBL thickness calculated using Equation (2) (Table 3). The values are determined 55–60 s after switching on the electric current. The determination procedure is illustrated in Figure 2.

The results of an average EC vortex zone thickness, d_ec_, determination in the studied range of current densities are summarized in Figure 12. These data were obtained using video frames in the range of 55–60 s from the moment the electric current was switched on. This time is sufficient for the systems under study to reach a stationary, or close to stationary, state. At *i/i*_lim_*^theor^* = 1.0, EC vortex clusters are not observed in a NaCl solution, likely due to their smallness. At the same time, at *i/i*_lim_*^theor^* = 2.0, EC vortex zone thickness is already equal to a half of the calculated thickness of the depleted DBL. The amplitude of PD oscillations in ChP for this current does not exceed 2 mV. With an increase in the current density, d_ec_ increases: at *i/i*_lim_*^theor^* = 6.0, EC vortex zone thickness is 2 times greater than *δ^theor^*. The amplitude of PD oscillations in the ChP stationary region approaches 100 mV, and their period is about 2–3 s (Figure 12).

In solutions of tartaric, phosphoric, and citric acid salts, large (more than 20 μm) vortex clusters are visualized starting from 3.5 (KHT), 4.3 (NaH_2_PO_4_), and 5.5 (NaH_2_Cit) *i/i*_lim_*^theor^*. The EC vortex zone thickness becomes comparable to the depleted DBL thickness at *i/i*_lim_*^theor^* equal to 2.9 (NaCl), 4.2 (KHT), 6.3 (NaH_2_PO_4_), and 7.9 (NaH_2_Cit). The observed shift in current densities at which *δ^theor^* ≤ d_ec_ is mainly caused by the higher rate of proton generation in systems that contain species of tartaric, phosphoric, and citric acids. The protons excluded from the AMX membrane into the depleted solution are highly mobile and cause a decrease in the electrical resistance of the depleted diffusion layer. Therefore, the threshold PD values required for the onset of the development of electroconvection by the EO II mechanism are achieved at higher currents as compared to the NaCl solution. 

## 4. Conclusions

Visualization of electroconvective vortices at the membrane surface and parallel measurements of the chronopotentiograms of this membrane are an informative way to study the mechanisms of mass transfer of species of polybasic weak acids. The dynamics of electroconvection development in solutions of salts of polybasic acids do not differ from those known for strong electrolytes. In particular, non-equilibrium electroconvection (electroosmosis of the second kind) develops in a threshold mode at times corresponding to a potential drop higher than that related to the transition time. More intense generation of protons in membrane systems containing polybasic acid anions suppresses electroconvection in comparison with NaCl solution. EC decreases in the order NaCl > NaHT > NaH_2_PO_4_ > NaH_2_Cit.

The EC vortex zone thickness increases with increasing current density and becomes comparable to the depleted DBL thickness at *i/i*_lim_*^theor^* equal to 2.9 (NaCl), 4.2 (KHT), 6.3 (NaH_2_PO_4_), and 7.9 (NaH_2_Cit). In all the solutions studied, the zones of EC vortex localization and, accordingly, the lowest electrolyte concentration, are situated in the “valleys” of the undulated AMX surface. 

To obtain more information on the interaction of the proton generation and EC in membrane systems containing weak polybasic acids, a non-stationary mathematical model is required. Such a model will be developed in the future.

## Figures and Tables

**Table 1:** Molar fraction of polybasic acid species at a given pH values of 0.02 M solutions.

Solution under Study		Mole Fraction of Polybasic Acid Species, %					
Designation	pH	H_2_A	HA^−^	A^2−^	H_3_A	H_2_A^−^	HA^2−^
NaCl	5.7 ± 0.1	-	-	-	-	-	-
NaH_2_PO_4_	4.6 ± 0.1	-	-	-	0.35	99.40	0.25
NaH_2_Cit	4.0 ± 0.1	-	-	-	10.30	76.40	13.30
NaHT	3.7 ± 0.1	12.2	68.5	19.3	-	-	-

**Table 2:** Some characteristics of the four-compartment electrodialysis flow cell.

Polarized (by electric current) membrane area, *S*, cm^2^	0.26
Intermembrane distance, *h*, cm	0.32
Polarized (by electric current) path length for solution in the desalination compartment, *L*, cm	0.53
Average linear solution flow velocity, *V*_0_, cm/s	0.07
Distance from the tip of the Luggin capillary to the surface of the AMX membrane, cm	0.15

**Table 3:** The diffusion boundary layer thickness, as well as the limiting current densities, calculated by Equations (1)–(4) and using experimental CVCs.

Electrolyte	*δ^theor^*, μm	*i*_lim_*^theor^*, mA/cm^2^	*i*_lim 1_*^exp^*, mA/cm^2^	*i*_lim 2_*^exp^*, mA/cm^2^
NaCl	231	3.39	3.38	-
NaHT	207	2.01	1.00	3.78
NaH_2_PO_4_	205	1.85	1.15	4.10
NaH_2_Cit	202	1.74	-	4.25

**Table 4:** Proton transport numbers in a depleted solution adjacent to the AMX surface, *T_s_^H+^*. Measurements made by Rybalkina and colleagues in 0.02 M electrolyte solutions at *i*/*i*_lim_*^theor^* = 1.5.

Electrolyte	*T_s_^H+^*
NaCl	0.11 ± 0.03
NaHT	0.22 ± 0.03
NaH_2_PO_4_	0.38 ± 0.03
NaH_2_Cit	0.57 ± 0.03

**Table 5:** Conductivity (ᴂ), surface resistance (*R*), and ohmic PD of AMX membranes (Δφ_Ω_) in 0.02 eq dm^−3^ solutions. The current densities (*i/i*_lim_*^Lev^* = 4.2 ± 0.4) correspond to the ChPs shown in the Figure 6, Figure 7, Figure 8 and Figure 9.

Electrolyte	Solution pH	Predominant Anion	ᴂ, mS cm^−1^	*R*, Ω cm^2^	*i*, mA cm^2^	Δφ_Ω_, mV
NaCl	5.7 ± 0.1 *	Cl^−^	3.88	3.30	13.66	45
Na_x_H_(3−X)_PO_4_	4.6 ± 0.1 *	H_2_PO_4_^−^	1.78	7.11	7.96	57
	9.0 ± 0.1 **	HPO_4_^2−^	2.82	4.54		36
Na_x_H_(2−X)_T	3.7 ± 0.1 *	HT^−^	0.79	16.16	7.64	124
	7.0 ± 0.1 **	T^2−^	0.95	13.47		103
Na_x_H_(3−X)_Cit	4.0 ± 0.1 *	H_2_Cit^−^	0.13	96.73	8.00	774
	9.0 ± 0.1 **	Cit^3−^	0.24	53.13		425

* pH of the feed solution; ** pH providing predominantly doubly charged anions of tartaric or phosphoric acid, as well as triply charged anions of citric acid in the solution and in the membrane.

## Supplementary Materials

The following are available online at https://www.mdpi.com/article/10.3390/ijms222413518/s1.

## References

[1] Robles Á., Aguado D., Barat R., Borrás L., Bouzas A., Giménez J.B., Martí N., Ribes J., Ruano M.V., Serralta J. (2020). New frontiers from removal to recycling of nitrogen and phosphorus from wastewater in the Circular Economy. Bioresour. Technol..

[2] Li X., Shen S., Xu Y., Guo T., Dai H., Lu X. (2021). Application of membrane separation processes in phosphorus recovery: A review. Sci. Total Environ..

[3] Mohammadi R., Tang W., Sillanpää M. (2021). A systematic review and statistical analysis of nutrient recovery from municipal wastewater by electrodialysis. Desalination.

[4] Merkel A., Voropaeva D., Fárová H., Yaroslavtsev A. (2020). High effective electrodialytic whey desalination at high temperature. Int. Dairy J..

[5] Sun X., Lu H., Wang J. (2017). Recovery of citric acid from fermented liquid by bipolar membrane electrodialysis. J. Clean. Prod..

[6] Khadem Modarresi Z., Mowla D., Karimi G. (2021). Electrodialytic separation of phosphate from sewage sludge ash using electrospun ion exchange membranes. Sep. Purif. Technol..

[7] Kattan Readi O.M., Rolevink E., Nijmeijer K. (2014). Mixed matrix membranes for process intensification in electrodialysis of amino acids. J. Chem. Technol. Biotechnol..

[8] Mikhaylin S., Patouillard L., Margni M., Bazinet L. (2018). Milk protein production by a more environmentally sustainable process: Bipolar membrane electrodialysis coupled with ultrafiltration. Green Chem..

[9] Chen G., Song W., Qi B., Li J., Ghosh R., Wan Y. (2015). Separation of protein mixtures by an integrated electro-ultrafiltration-electrodialysis process. Sep. Purif. Technol..

[10] Teixeira A., Baenas N., Dominguez-Perles R., Barros A., Rosa E., Moreno D.A., Garcia-Viguera C. (2014). Natural bioactive compounds from winery by-products as health promoters: A review. Int. J. Mol. Sci..

[11] Conidi C., Drioli E., Cassano A. (2018). Membrane-based agro-food production processes for polyphenol separation, purification and concentration. Curr. Opin. Food Sci..

[12] Rotta E.H., Bitencourt C.S., Marder L., Bernardes A.M. (2019). Phosphorus recovery from low phosphate-containing solution by electrodialysis. J. Memb. Sci..

[13] Wang Q., Chen G.Q., Lin L., Li X., Kentish S.E. (2021). Purification of organic acids using electrodialysis with bipolar membranes (EDBM) combined with monovalent anion selective membranes. Sep. Purif. Technol..

[14] Chandra A., Tadimeti J.G.D., Bhuvanesh E., Pathiwada D., Chattopadhyay S. (2018). Switching selectivity of carboxylic acids and associated physico-chemical changes with pH during electrodialysis of ternary mixtures. Sep. Purif. Technol..

[15] (2016). Saltworks awarded funding to commercialize Ammonia Splitter. Filtr. Ind. Anal..

[16] Vital B., Torres E.V., Sleutels T., Gagliano M.C., Saakes M., Hamelers H.V.M. (2021). Fouling fractionation in reverse electrodialysis with natural feed waters demonstrates dual media rapid filtration as an effective pre-treatment for fresh water. Desalination.

[17] Wang Y., Zhang N., Huang C., Xu T. (2011). Production of monoprotic, diprotic, and triprotic organic acids by using electrodialysis with bipolar membranes: Effect of cell configurations. J. Memb. Sci..

[18] Hülber-Beyer É., Bélafi-Bakó K., Nemestóthy N. (2021). Low-waste fermentation-derived organic acid production by bipolar membrane electrodialysis—An overview. Chem. Pap..

[19] Sarapulova V., Nevakshenova E., Pismenskaya N., Dammak L., Nikonenko V. (2015). Unusual concentration dependence of ion-exchange membrane conductivity in ampholyte-containing solutions: Effect of ampholyte nature. J. Memb. Sci..

[20] Belashova E.D., Pismenskaya N.D., Nikonenko V.V., Sistat P., Pourcelly G. (2017). Current-voltage characteristic of anion-exchange membrane in monosodium phosphate solution. Modelling and experiment. J. Memb. Sci..

[21] Liu J., Liang J., Feng X., Cui W., Deng H., Ji Z., Zhao Y., Guo X., Yuan J. (2021). Effects of inorganic ions on the transfer of weak organic acids and their salts in electrodialysis process. J. Memb. Sci..

[22] Chandra A., Tadimeti J.G.D., Chattopadhyay S. (2018). Transport hindrances with electrodialytic recovery of citric acid from solution of strong electrolytes. Chin. J. Chem. Eng..

[23] Martí-Calatayud M.C., Evdochenko E., Bär J., García-Gabaldón M., Wessling M., Pérez-Herranz V. (2020). Tracking homogeneous reactions during electrodialysis of organic acids via EIS. J. Memb. Sci..

[24] Rybalkina O., Tsygurina K., Melnikova E., Mareev S., Moroz I., Nikonenko V., Pismenskaya N. (2019). Partial fluxes of phosphoric acid anions through anion-exchange membranes in the course of NaH2PO4 solution electrodialysis. Int. J. Mol. Sci..

[25] Barros K.S., Espinosa D.C.R. (2018). Chronopotentiometry of an anion-exchange membrane for treating a synthesized free-cyanide effluent from brass electrodeposition with EDTA as chelating agent. Sep. Purif. Technol..

[26] Gally C., García-Gabaldón M., Ortega E.M., Bernardes A.M., Pérez-Herranz V. (2020). Chronopotentiometric study of the transport of phosphoric acid anions through an anion-exchange membrane under different pH values. Sep. Purif. Technol..

[27] Vobecká L., Svoboda M., Beneš J., Belloň T., Slouka Z. (2018). Heterogeneity of heterogeneous ion-exchange membranes investigated by chronopotentiometry, and X-ray computed microtomography. J. Memb. Sci..

[28] Barros K.S., Martí-Calatayud M.C., Scarazzato T., Bernardes A.M., Espinosa D.C.R., Pérez-Herranz V. (2021). Investigation of ion-exchange membranes by means of chronopotentiometry: A comprehensive review on this highly informative and multipurpose technique. Adv. Colloid Interface Sci..

[29] Roghmans F., Evdochenko E., Martí-Calatayud M.C., Garthe M., Tiwari R., Walther A., Wessling M. (2020). On the permselectivity of cation-exchange membranes bearing an ion selective coating. J. Memb. Sci..

[30] Chandra A., Chattopadhyay S. (2019). Physicochemical interactions of organic acids influencing microstructure and permselectivity of anion exchange membrane. Colloids Surf. A Physicochem. Eng. Asp..

[31] Chandra A., Chattopadhyay S. (2020). Chain length and acidity of carboxylic acids influencing adsorption/desorption mechanism and kinetics over anion exchange membrane. Colloids Surf. A Physicochem. Eng. Asp..

[32] Beaulieu M., Perreault V., Mikhaylin S., Bazinet L. (2020). How overlimiting current condition influences lactic acid recovery and demineralization by electrodialysis with nanofiltration membrane: Comparison with conventional electrodialysis. Membranes.

[33] Kozaderova O.A., Niftaliev S.I., Kim K.B. (2018). Ionic Transport in Electrodialysis of Ammonium Nitrate. Russ. J. Electrochem..

[34] Martí-Calatayud M., García-Gabaldón M., Pérez-Herranz V. (2018). Mass Transfer Phenomena during Electrodialysis of Multivalent Ions: Chemical Equilibria and Overlimiting Currents. Appl. Sci..

[35] Scarazzato T., Panossian Z., García-Gabaldón M., Ortega E.M., Tenório J.A.S., Pérez-Herranz V., Espinosa D.C.R. (2017). Evaluation of the transport properties of copper ions through a heterogeneous ion-exchange membrane in etidronic acid solutions by chronopotentiometry. J. Memb. Sci..

[36] Simons R. (1984). Electric field effects on proton transfer between ionizable groups and water in ion exchange membranes. Electrochim. Acta.

[37] Tanioka A., Kawaguchi M., Hamada M., Yoshie K. (1998). Dissociation Constant of a Weak Electrolyte in Charged Membrane. J. Phys. Chem. B.

[38] Liu G., Wu D., Chen G., Halim R., Liu J., Deng H. (2021). Comparative study on tartaric acid production by two-chamber and three-chamber electro-electrodialysis. Sep. Purif. Technol..

[39] Levich V.G. (1962). Physicochemical Hydrodynamics.

[40] Nikonenko V.V., Mareev S.A., Pis’menskaya N.D., Uzdenova A.M., Kovalenko A.V., Urtenov M.K., Pourcelly G. (2017). Effect of electroconvection and its use in intensifying the mass transfer in electrodialysis (Review). Russ. J. Electrochem..

[41] Bazinet L., Geoffroy T.R. (2020). Electrodialytic Processes: Market Overview, Membrane Phenomena, Recent Developments and Sustainable Strategies. Membranes.

[42] Mani A., Wang K.M. (2020). Electroconvection Near Electrochemical Interfaces: Experiments, Modeling, and Computation. Annu. Rev. Fluid Mech..

[43] Dukhin S.S. (1991). Electrokinetic phenomena of the second kind and their applications. Adv. Colloid Interface Sci..

[44] Mishchuk N.A. (2010). Concentration polarization of interface and non-linear electrokinetic phenomena. Adv. Colloid Interface Sci..

[45] Rubinstein I., Zaltzman B. (2015). Equilibrium Electroconvective Instability. Phys. Rev. Lett..

[46] Cortelezzi L., Karagozian A.R. (2001). On the formation of the counter-rotating vortex pair in transverse jets. J. Fluid Mech..

[47] del Álamo J.C., Jiménez J., Zandonade P., Moser R.D. (2006). Self-similar vortex clusters in the turbulent logarithmic region. J. Fluid Mech..

[48] Chang H.-C., Demekhin E.A., Shelistov V.S. (2012). Competition between Dukhin’s and Rubinstein’s electrokinetic modes. Phys. Rev. E.

[49] Wenten I.G., Khoiruddin K., Alkhadra M.A., Tian H., Bazant M.Z. (2020). Novel ionic separation mechanisms in electrically driven membrane processes. Adv. Colloid Interface Sci..

[50] Mikhaylin S., Nikonenko V., Pismenskaya N., Pourcelly G., Choi S., Kwon H.J., Han J., Bazinet L. (2016). How physico-chemical and surface properties of cation-exchange membrane affect membrane scaling and electroconvective vortices: Influence on performance of electrodialysis with pulsed electric field. Desalination.

[51] Barros K.S., Scarazzato T., Pérez-Herranz V., Espinosa D.C.R. (2020). Treatment of Cyanide-Free Wastewater from Brass Electrodeposition with EDTA by Electrodialysis: Evaluation of Underlimiting and Overlimiting Operations. Membranes.

[52] Cecile Urbain Marie G., Perreault V., Henaux L., Carnovale V., Aluko R.E., Marette A., Doyen A., Bazinet L. (2019). Impact of a high hydrostatic pressure pretreatment on the separation of bioactive peptides from flaxseed protein hydrolysates by electrodialysis with ultrafiltration membranes. Sep. Purif. Technol..

[53] Güler E., van Baak W., Saakes M., Nijmeijer K. (2014). Monovalent-ion-selective membranes for reverse electrodialysis. J. Memb. Sci..

[54] Sarapulova V.V., Shkorkina I.V., Mareev S.A., Pismenskaya N.D., Kononenko N.A., Larchet C., Dammak L., Nikonenko V.V. (2019). Transport Characteristics of Fujifilm Ion-Exchange Membranes as Compared to Homogeneous Membranes AMX and CMX and to Heterogeneous Membranes MK-40 and MA-41. Membranes.

[55] Berezina N.P., Kononenko N.A., Dyomina O.A., Gnusin N.P. (2008). Characterization of ion-exchange membrane materials: Properties vs structure. Adv. Colloid Interface Sci..

[56] Lide D.R. (2005). CRC Handbook of Chemistry and Physics.

[57] Kwak R., Guan G., Peng W.K., Han J. (2013). Microscale electrodialysis: Concentration profiling and vortex visualization. Desalination.

[58] Magut P.K.S., Das S., Fernand V.E., Losso J., McDonough K., Naylor B.M., Aggarwal S., Warner I.M. (2013). Tunable Cytotoxicity of Rhodamine 6G via Anion Variations. J. Am. Chem. Soc..

[59] Newman J.S. (1973). Electrochemical Systems.

[60] Titorova V.D., Mareev S.A., Gorobchenko A.D., Gil V.V., Nikonenko V.V., Sabbatovskii K.G., Pismenskaya N.D. (2021). Effect of current-induced coion transfer on the shape of chronopotentiograms of cation-exchange membranes. J. Memb. Sci..

[61] Park S., Kwak R. (2020). Microscale electrodeionization: In situ concentration profiling and flow visualization. Water Res..

[62] Simons R. (1985). Water splitting in ion exchange membranes. Electrochim. Acta.

[63] Tanaka Y. (2002). Water dissociation in ion-exchange membrane electrodialysis. J. Memb. Sci..

[64] Zabolotskii V.I., Sharafan M.V., Shel’deshov N.V. (2012). The dissociation rate of water molecules in systems with cation-and anion-exchange membranes. Russ. J. Electrochem..

[65] Aristov I.V., Bobreshova O.V., Kulintsov P.I. (2001). Transfer of amino acids through a membrane/solution interface in the presence of heterogeneous chemical protonation reaction. Russ. J. Electrochem..

[66] Melnikova E.D., Pismenskaya N.D., Bazinet L., Mikhaylin S., Nikonenko V.V. (2018). Effect of ampholyte nature on current-voltage characteristic of anion-exchange membrane. Electrochim. Acta.

[67] Pismenskaya N.D., Rybalkina O.A., Kozmai A.E., Tsygurina K.A., Melnikova E.D., Nikonenko V.V. (2020). Generation of H+ and OH− ions in anion-exchange membrane/ampholyte-containing solution systems: A study using electrochemical impedance spectroscopy. J. Memb. Sci..

[68] Helfferich F.G. (1962). Ion Exchange.

[69] Kharkats Y.I. (1985). The mechanism of “supralimiting” currents at ion-exchange membrane/electrolyte interfaces. Sov. Electrochem..

[70] Shelistov V.S., Demekhin E.A., Ganchenko G.S. (2014). Electrokinetic instability near charge-selective hydrophobic surfaces. Phys. Rev. E.

[71] Choi J.-H., Lee H.-J., Moon S.-H. (2001). Effects of Electrolytes on the Transport Phenomena in a Cation-Exchange Membrane. J. Colloid Interface Sci..

[72] Rubinstein I., Zaltzman B. (2007). Electro-convective versus electroosmotic instability in concentration polarization. Adv. Colloid Interface Sci..

[73] Barros K.S., Ortega E.M., Pérez-Herranz V., Espinosa D.C.R. (2020). Evaluation of brass electrodeposition at RDE from cyanide-free bath using EDTA as a complexing agent. J. Electroanal. Chem..

[74] Korzhova E., Pismenskaya N., Lopatin D., Baranov O., Dammak L., Nikonenko V. (2016). Effect of surface hydrophobization on chronopotentiometric behavior of an AMX anion-exchange membrane at overlimiting currents. J. Memb. Sci..

[75] Krol J. (1999). Chronopotentiometry and overlimiting ion transport through monopolar ion exchange membranes. J. Memb. Sci..

[76] Belashova E.D., Kharchenko O.A., Sarapulova V.V., Nikonenko V.V., Pismenskaya N.D. (2017). Effect of Protolysis Reactions on the Shape of Chronopotentiograms of a Homogeneous Anion-Exchange Membrane in NaH2PO4 Solution. Pet. Chem..

[77] Kumar P., Rubinstein S.M., Rubinstein I., Zaltzman B. (2020). Mechanisms of hydrodynamic instability in concentration polarization. Phys. Rev. Res..

[78] Vasil’eva V.I., Shaposhnik V.A., Grigorchuk O.V., Petrunya I.P. (2006). The membrane–solution interface under high-performance current regimes of electrodialysis by means of laser interferometry. Desalination.

[79] Shaposhnik V.A., Vasil’eva V.I., Grigorchuk O.V. (2008). The interferometric investigations of electromembrane processes. Adv. Colloid Interface Sci..

[80] Urtenov M.K., Uzdenova A.M., Kovalenko A.V., Nikonenko V.V., Pismenskaya N.D., Vasil’eva V.I., Sistat P., Pourcelly G. (2013). Basic mathematical model of overlimiting transfer enhanced by electroconvection in flow-through electrodialysis membrane cells. J. Memb. Sci..

[81] de Valença J., Jõgi M., Wagterveld R.M., Karatay E., Wood J.A., Lammertink R.G.H. (2018). Confined Electroconvective Vortices at Structured Ion Exchange Membranes. Langmuir.

[82] Rybalkina O.A., Moroz I.A., Gorobchenko A.D., Pismenskaya N.D., Nikonenko V.V. (2022). Razvitie elektrokonvektsii u volnistoy poverhnosti anionoobmennoy membrany v rastvorah hlorida i gidrotartrata natriya. Membr. Membr. Technol..

[83] Malek P., Ortiz J.M., Richards B.S., Schäfer A.I. (2013). Electrodialytic removal of NaCl from water: Impacts of using pulsed electric potential on ion transport and water dissociation phenomena. J. Memb. Sci..

[84] Zabolotskii V.I., Bugakov V.V., Sharafan M.V., Chermit R.K. (2012). Transfer of electrolyte ions and water dissociation in anion-exchange membranes under intense current conditions. Russ. J. Electrochem..

[85] Slouka Z., Senapati S., Yan Y., Chang H.-C. (2013). Charge Inversion, Water Splitting, and Vortex Suppression Due to DNA Sorption on Ion-Selective Membranes and Their Ion-Current Signatures. Langmuir.

[86] Andersen M.B., van Soestbergen M., Mani A., Bruus H., Biesheuvel P.M., Bazant M.Z. (2012). Current-Induced Membrane Discharge. Phys. Rev. Lett..

[87] Stockmeier F., Schatz M., Habermann M., Linkhorst J., Mani A., Wessling M. (2021). Direct 3D observation and unraveling of electroconvection phenomena during concentration polarization at ion-exchange membranes. J. Memb. Sci..

[88] Tsai P., Peters A.M., Pirat C., Wessling M., Lammertink R.G.H., Lohse D. (2009). Quantifying effective slip length over micropatterned hydrophobic surfaces. Phys. Fluids.

