# Supplementary material for: Influence of Electroconvection on Chronopotentiograms of an Anion-Exchange Membrane in Solutions of Weak Polybasic Acid Salts
Source: Int J Mol Sci. 2021 Dec 16;22(24):13518. doi: 10.3390/ijms222413518 (PMC8708104; doi:10.3390/ijms222413518)
Supplement: Supplementary file 1 [file ijms-22-13518-s001.zip › Supplementary Materials.pdf]

Influence of electroconvection on chronopotentiograms of anion-exchange membrane in solutions of weak polybasic acid salts

Natalia Pismenskaya, Olesya Rybalkina, Ilya Moroz, Semen Mareev, Victor Nikonenko

Kuban State University, 149, Stavropolskaya Str., Krasnodar, 350040, Russian Federation; n_pismen@mail.ru (N.P.); olesia93rus@mail.ru (O.R.); ilya_moroz@mail.ru (I.M.); v_nikonenko@mail.ru (V.N.);

* Correspondence: v_nikonenko@mail.ru; Tel.: +7-918-41-45-816

Supplementary materials

S1. Membranes

Homogeneous anion exchange AMX membrane with quaternary ammonium bases as fixed functional groups and small amounts of weakly basic primary and secondary amines is made by the paste method: a reinforcing polyvinyl chloride (PVC) fabric (Figure S1a) is introduced into the membrane at the step of producing a composite material from a paste, which consists of a PVC powder and the monomers styrene and divinylbenzene [1]. When these monomers are copolymerized, a composite ion-exchange material is formed, in which PVC particles are incorporated; the diameter of these particles does not exceed 100 nm (Figure S1a). There is strong adhesion between the ion exchange material and the reinforcing cloth, which is due to the fact that PVC is included in both materials. This preparation procedure eliminates the possibility of the formation of macropores (larger than 200 nm) in the bulk of homogeneous membranes. Although such membranes are called homogeneous [2,3], their structure is heterogeneous at the nanoscale and includes two different polymer materials, as well as reinforcing cloth, with fibers about 30 µm in diameter (Figure S1b). AMX membrane has undulated surface (Figure S2). The distance between the levels corresponding to the top of the “hills” and the bottom of the “valleys” in swollen state is equal to 30±µm [4].

Heterogeneous cation exchange MK-40 membrane is made by hot rolling of milled cation-exchange resins KU-2-8, as well as a high-density polyethylene powder. Then reinforcing nylon mesh is introduced (Figure 1d) using the hot pressing method [5]. The size of ion-exchange resin particles ranges from 5 to 50 µm. The adhesion between individual resin particles, polyethylene, and the reinforcing cloth is low, resulting in gaps that form macropores (about 1 µm in size) when the membrane contacts solutions [6]. The resin particles are evenly distributed throughout the IEM (Figure 1c); their tops extend beyond the polyethylene coated surface of heterogeneous membranes (Figure 1c). The resin is a copolymer of styrene and

divinylbenzene (8%). It contains sulphonate fixed groups (KU-2-8). Some of the characteristics of the membranes under study are presented in Table S1.

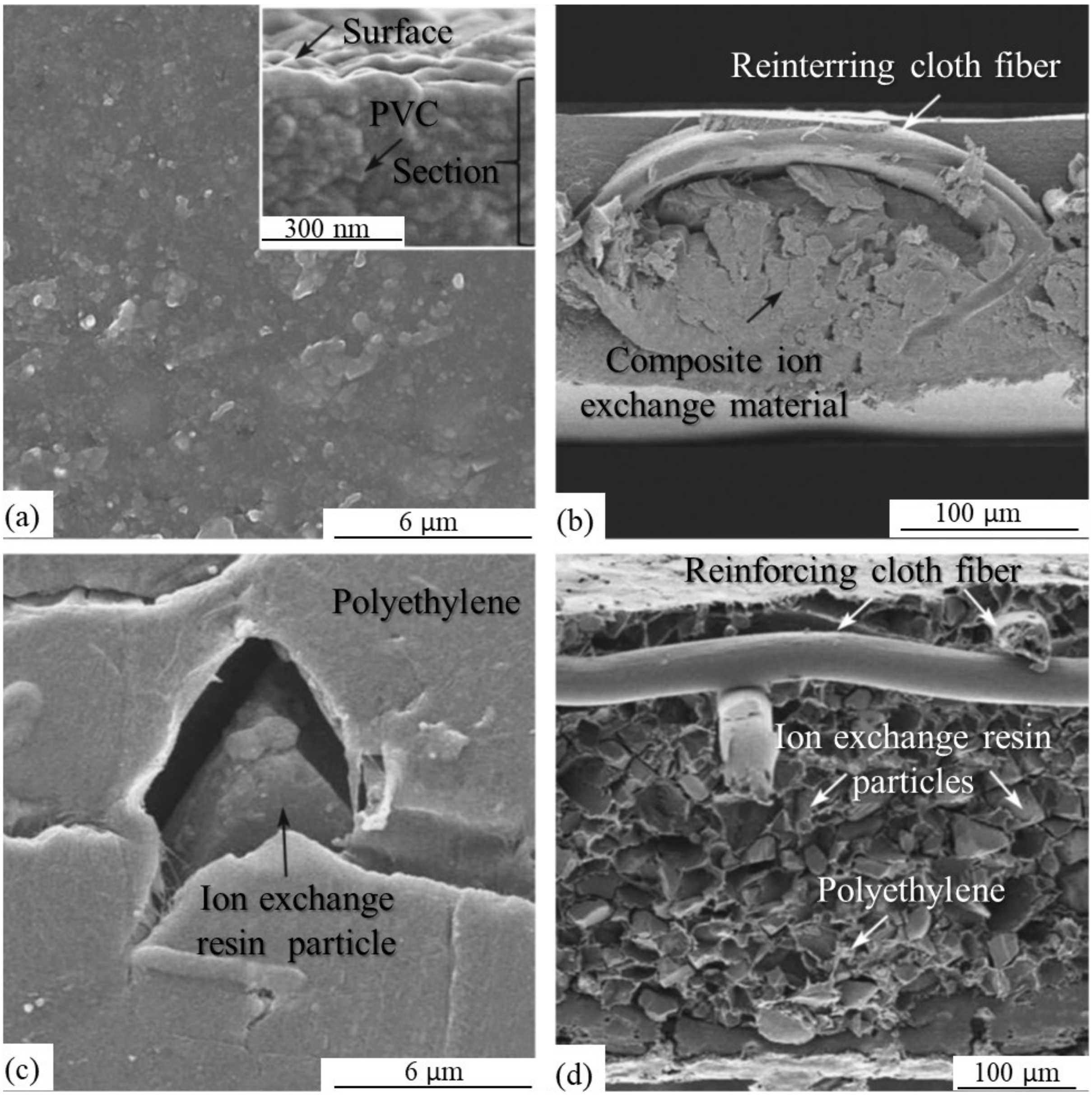

Figure S1. SEM images of (a, c) surfaces and (b, d) sections of (a, b) a homogeneous AMX membrane and (c, d) a heterogeneous MK-40 membrane (d). The heterogeneous membrane MA-41 has a structure similar to that of MK-40.

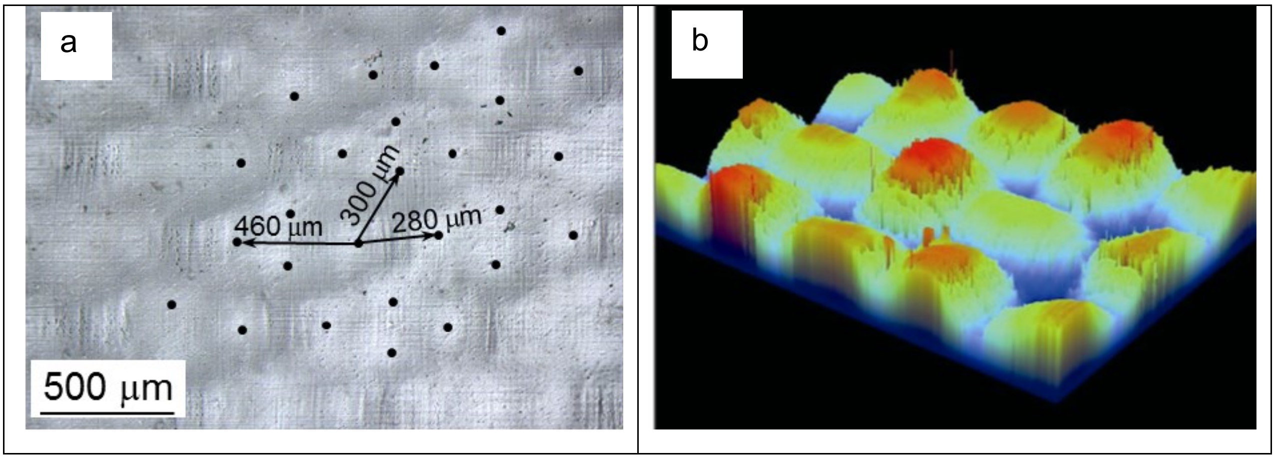

Figure S2. Optical image of the surfaces of the swollen membranes AMX [7] (a) and image of a dry AMX membrane obtained using optical interferometry [7]. The tops of the hills in Figure (a) are denoted by black dot.

Table S1

Some characteristics of a homogeneous Neosepta AMX (Astom, Japan) membrane under study and a heterogeneous MK-40, MA-41 (Shchekinoazot, Russia) membranes, which was used as an auxiliary membrane [8].

	AMX	MK-40
The thickness in 0.02 M NaCl solution, µm	140±3	520±20
The exchange capacity in the swollen state, mol dm^{-3}	1.22±0.05	1.52 ± 0.08
Water content of swollen membrane equilibrated with 0.02 eq dm^{-3} NaCl solution, %	16 ± 2	30 ± 2
Fraction of the conducting surface, Θ [9]	1.0	0.22 ±0.03
Contact angle in the swollen (0.02 M NaCl solution) state, grad	62 [7]	55±2 [10]

S2 Solutions

Monosodium phosphate (NaH_2PO_4) is a salt of the tribasic orthophosphoric acid. The latter has the following structure

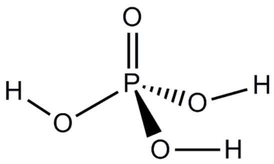

Hydrogen tartrate ion, $C_4H_5O_6^-$, denoted by HT^-, is a singly charged anion of the tartaric acid (H_2T). The IUPAC name of H_2T is 2,3 - dihydroxybutanedioic acid, $C_4H_6O_6$, which has the following structure

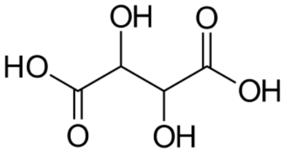

Dihydrogen citrate ion, $C_6H_7O_7^-$, denoted by H_2Cit^- is the salt of the tribasic citric acid, which preferred IUPAC name is 2-hydroxypropane-1,2,3-tricarboxylic acid. The structural formula of the citric acid is:

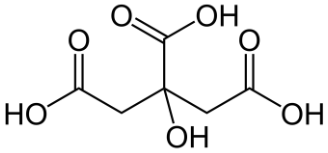

The proton-transfer reactions between water molecules and polybasic acid species (the cases of phosphoric and citric acids with general formula H_nA, where the maximum value of n is 3) are presented as follows:

$$H_2A^- + H_2O \underset{k_{-1}}{\overset{k_1}{\rightleftarrows}} H_3A + OH^- ; \qquad K_{b1} = \frac{c_{H_3A}c_{OH^-}}{c_{H_2A^-}} \tag{S1}$$

$$H_3A + H_2O \underset{k_{-2}}{\overset{k_2}{\rightleftarrows}} H_2A^- + H_3O^+ ; \qquad K_{a1} = \frac{c_{H_2A^-}c_{H^+}}{c_{H_3A}} \tag{S2}$$

$$HA^{2-} + H_2O \underset{k_{-3}}{\overset{k_3}{\rightleftarrows}} H_2A^- + OH^- ; \qquad K_{b2} = \frac{c_{H_2A^-}c_{OH^-}}{c_{HA^{2-}}} \tag{S3}$$

$$H_2A^- + H_2O \underset{k_{-4}}{\overset{k_4}{\rightleftarrows}} HA^{2-} + H_3O^+ ; \qquad K_{a2} = \frac{c_{HA^{2-}}c_{H^+}}{c_{H_2A^-}} \tag{S4}$$

$$A^{3-} + H_2O \underset{k_{-5}}{\overset{k_5}{\rightleftarrows}} HA^{2-} + OH^- ; \qquad K_{b3} = \frac{c_{HA^{2-}}c_{OH^-}}{c_{A^{3-}}} \tag{S5}$$

$$HA^{2-} + H_2O \underset{k_{-6}}{\overset{k_6}{\rightleftarrows}} A^{3-} + H_3O^+ ; \qquad K_{a3} = \frac{c_{A^{3-}}c_{H^+}}{c_{HA^{2-}}} \tag{S6}$$

Calculation of the mole fraction of ampholyte species in solution and membrane

Figure S2 shows the distribution of species of the polybasic acids under study (in mole fractions) vs. the pH of the solution. These distributions are calculated using the appropriate equilibrium equations and the pK_a values presented in Table S2.

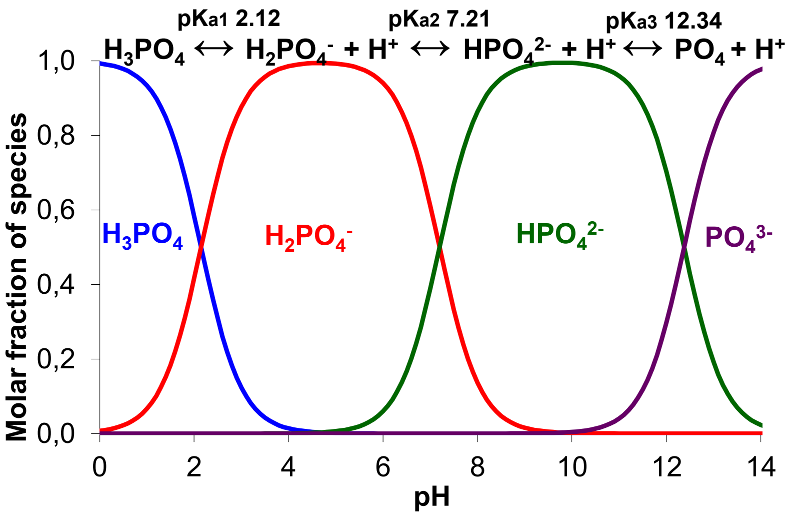

a

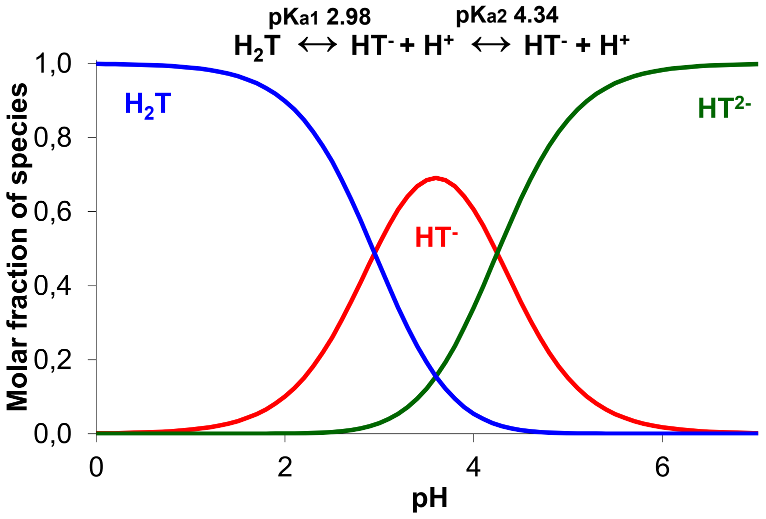

b

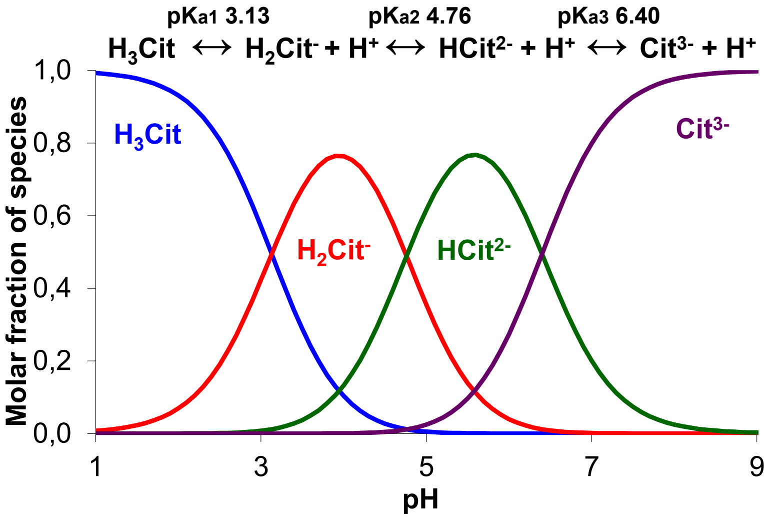

c

Figure S2. Speciation diagrams: distribution of the phosphoric (a), tartaric (b) and citric (c) acid species (in mole fractions) vs. the pH of the solution.

Calculation of the rate constants

There are relationships between the pseudo-unimolecular forward rate constants and backward rate constants, which involve the equilibrium constants, the acid dissociation (K_a) or the base ionization (K_b) constants. For example, in the case of reactions (S1) and (S2), we have [11,12]:

$$\frac{k_1'}{k_{-1}} = K_{b1} = \frac{K_w}{K_{a1}}; \qquad \frac{k_2'}{k_{-2}} = K_{a1} = \frac{K_w}{K_{b1}} \tag{S7}$$

where K_w (equal to 10^{-14} mol^2 dm^{-6}) is the water dissociation constant; K_{ai} (mol dm^{-3}) is defined for each step of dissociation i=1, 2, 3 by Eqs. (S2), (S4) and (S6), respectively; $K_{bi} = K_w / K_{ai}$. Equations, similar to Eqs. (S7), can also be written for the 2-nd and 3-rd dissociation steps. The pseudo-unimolecular forward rate constants k_1' and k_2' are obtained, when the concentration of water, c_{H_2O}, is considered as a constant whose value is taken into account in the value of these constants: $k_1' = k_1 c_{H_2O}$, $k_2' = k_2 c_{H_2O}$ [11,13].

The values of the dissociation constants, K_{ai}, used in the calculations are given in Table S2. The values of the backward rate constants k_{-1} and k_{-2} are both taken equal to 10^{10} dm^{-3} mol^{-1} s^{-1} [11].

Table S2

The acid dissociation constant, pK_{ai} (at 25 °C) of acids [14], and pseudo-unimolecular forward rate constants for the protonation (k'_1) and deprotonation (k'_2) reactions for different dissociation steps of the orthophosphoric, tartaric and citric acids.

	pK_{ai} (mol L^{-1})			k'_1 and k'_2, s^{-1}			
	H_3PO_4	***H_3Cit***	***H_2T***		***H_3PO_4***	***H_3Cit***	***H_2T***
**1-st step**	2.12	3.13	2.98	k'_1	$1\cdot10^{2}$	$1\cdot10^{-1}$	$1\cdot10^{-1}$
				k'_2	$8\cdot10^{7}$	$7\cdot10^{6}$	$1\cdot10^{7}$
**2-nd step**	7.21	4.76	4.34	k'_1	$2\cdot10^{3}$	6	2
				k'_2	$6\cdot10^{2}$	$2\cdot10^{5}$	$5\cdot10^{5}$
**3-rd step**	12.34	6.40	-	k'_1	$2\cdot10^{8}$	$3\cdot10^{2}$	-
				k'_2	$5\cdot10^{-3}$	$4\cdot10^{3}$	-

S3. Theory

S3.1. The Lévêque limiting current density and diffusion layer thickness.

S3.1.1. The case of a binary electrolyte (NaCl, pH 5.7±0.1, NaH_2PO_4, pH 4.6±0.1)

The Leveque equation [(S8) and equation (S9)] are used to estimate the theoretical limiting current, i_{lim}^{Lev}, and the thickness of the depleted diffusion layer, δ^{Lev}:

$$i_{\lim}^{Lev} = \frac{z_1 F D c_1^0}{h(T_1 - t_1)}\left[1.47\left(\frac{h^2 V_0}{LD}\right)^{1/3} - 0.2\right] \tag{S8}$$

$$\delta^{Lev} = 0.71h\left(\frac{LD}{h^2 V_0}\right)^{1/3} \tag{S9}$$

Here z_1 is the charge number of counterion 1, F is Faraday constant, D и t_1 are the diffusion coefficient of the electrolyte and the electromigration transfer number of the counterion at infinite dilution of the solution. The transport number of counterions in the membrane, T_1, was considered equal to one; c_1 is its molar concentration in the feed solution entering the DC, V_0 is the average linear velocity of the solution flowing between the membranes forming the DC, h is the distance between the membranes, L is the length of the channel. These equations were obtained for the diffusion-convection heat transfer [15] and after were adapted to diffusion-convection mass transfer in electrode [16] and ion exchange membrane [17] systems. For 1: 1 electrolyte and laminar hydrodynamic regime. Note that the value 0.71 for the factor in the right-hand part of Eq. (S9) is given by the numerical solution of the 2D convection-diffusion problem whose asymptotic solution is expressed by Eq. (S8). However, often [18,19] Eq. (S9) is used with a factor 0.68 (Eq. (6) in the main text), which is obtained using the Peers equation [19] and the approximation of Eq. (S8), where the second term in the brackets is neglected (Eq. (5) in the main text). It is worth noting that this equation is applicable only for relatively short channel lengths ($L \le 0.02h^2 V_0 / D$) [17].

S3.1.2. The case of a ternary electrolyte (NaH_2Cit, pH 4.6±0.1; NaHT or KHT, pH 3.7±0.1)

Determination of ionic concentrations in an ampholyte solution

Taking into account all cations and anions, the electroneutrality condition for an external solution can be written as follows:

$$C_{H_2A^-} + 2C_{HA^{2-}} + 3C_{A^{3-}} = C_{Na^+} + C_{H^+} \tag{S10}$$

The solutions used had pH<6; therefore, we neglected the concentration of OH^- ions.

Since solutions of a predetermined concentration are used during the experiment, the C_{Na^+} value is known. In addition, the pH of the solution can be measured and the concentration of H^+ ions (C_{H^+}) can be determined. Thus, the parameters $C_{H_2A^-}$, $C_{HA^{2-}}$, $C_{A^{3-}}$ remain unknown in Eq. (S10). These values can be calculated from the equations derived from Eqs. (S1) - (S6):

$$C_{H^+} = 10^{-pH} \quad (S11)$$

$$C_{H_2A^-} = \frac{K_{a1}C_{H_3A}}{C_{H^+}} \quad (S12)$$

$$C_{HA^{2-}} = \frac{K_{a2}C_{H_2A^-}}{C_{H^+}} \quad (S13)$$

$$C_{A^{3-}} = \frac{K_{a3}C_{HA^{2-}}}{C_{H^+}} \quad (S14)$$

Expressions (S12) - (S14) allow the concentration of all anions to be expressed in terms of C_{H_3A}. After substituting the corresponding equations in Eq. (S10), there will be only one variable, C_{H_3A}. Using the function "Search for a solution" in Excel, we select such a value for this parameter that the left side of the Eq. (S10) differs from the right side by no more than 0.001.

Determination of ionic concentrations in an anion-exchange membrane contacting with an ampholyte solution

For an anion-exchange membrane, the electroneutrality condition is written for the internal solution filling the pores. The concentration of H^+, since it is a co-ion, is negligible and is not taken into account in Eq. (S15). In addition, the Na^+ ion is also a co-ion for the anion-exchange membrane, so the C_{Na^+} value can be neglected.

$$\bar{C}_{H_2A^-} + 2\bar{C}_{HA^{2-}} + 3\bar{C}_{A^{3-}} + \bar{C}_{OH^-} = \bar{C}_{Na^+} + \bar{C}_x \quad (S15)$$

Here $\bar{C}_x$ is the concentration of charged fixed groups, its value can be found using the following expression:

$$\bar{C}_x = \frac{Q}{p} \quad (S16)$$

where p is the membrane porosity, which is expressed as the water content in cm^3 H_2O / cm^3 of the wet membrane.

The ion concentrations inside the membrane are found using the equations:

$$\bar{C}_{H^+} = \frac{K_w}{\bar{C}_{OH^-}} \quad (S17)$$

$$\bar{C}_{H_2A^-} = \frac{C_{H_2A^-}\bar{C}_{OH^-}}{K^{OH^-}_{H_2A^-}C_{OH^-}} \quad (S18)$$

$$\overline{C}_{HA^{2-}} = \frac{K_{a2}\overline{C}_{H_2A^-}}{C_{H^+}} \tag{S19}$$

$$\overline{C}_{A^{3-}} = \frac{K_{a3}\overline{C}_{HA^{2-}}}{C_{H^+}} \tag{S20}$$

where $K_{H_2A^-}^{OH^-}$ is the constant of ion exchange equilibrium between H_2A^- and OH^-.

The equation for $\overline{C}_{H^+}$ is substituted into Eqs. (S19) and (S20). Expressions (S18) - (S20) can be substituted into Eq. (S15). After that, there will be only one unknown quantity, $\overline{C}_{OH^-}$. Using the function "Find a solution" in Excel, we select such a value for this parameter that the left side of Eq. (S15) differs from the right side by no more than 0.001.

The Lévêque limiting current density in a mixed electrolyte solution

Let us consider a ternary electrolyte composed of two kinds of counterions, 1 and 2, and one kind of coion, a later on, we consider a solution of this ternary electrolyte in a diffusion layer adjacent to an ion-exchange membrane.

The Nernst-Planck equations for these ions read:

$$j_1 = -D_1\left(\frac{dc_1}{dx} + z_1c_1\frac{F}{RT}\frac{d\varphi}{dx}\right) \tag{S21}$$

$$j_2 = -D_2\left(\frac{dc_2}{dx} + z_2c_2\frac{F}{RT}\frac{d\varphi}{dx}\right) \tag{S22}$$

$$j_A = -D_A\left(\frac{dc_A}{dx} + z_Ac_A\frac{F}{RT}\frac{d\varphi}{dx}\right) \tag{S23}$$

After dividing each of the equations (S21)-(S23) by D_i, summing the results, and taking into account the electroneutrality condition

$$z_1c_1 + z_2c_2 + z_Ac_A = 0 \tag{S24}$$

we find:

$$\frac{j_1}{D_1} + \frac{j_2}{D_2} + \frac{j_A}{D_A} = -\frac{d(c_1+c_2+c_3)}{dx} = -\frac{(1+|z_1/z_A|)dc_1}{dx} - \frac{(1+|z_2/z_A|)dc_2}{dx} \tag{S25}$$

The last equality in Eq. (S25) is obtained after eliminating c_A using Eq. (S24). Since j_i do not change along the coordinate x in a stationary state, Eq. (S25) can be easily integrated over the thickness of the diffusion layer. If the current density is equal to the limiting one, the concentrations of all ions at the membrane surface are very close to zero. In this case, we can write:

$$\frac{j_{1\lim}^{theor}}{D_1}+\frac{j_{2\lim}^{theor}}{D_2}=\frac{\left(1+\left|z_1/z_A\right|\right)c_1^0}{\delta}+\frac{\left(1+\left|z_2/z_A\right|\right)c_2^0}{\delta}-\frac{j_{A\lim}^{theor}}{D_A} \quad \text{(S26)}$$

It follows from Eq. (S16) that the limiting flux density of counterion i can be represented as [20]:

$$j_{i\lim}^{theor}=\frac{\left(1+\left|z_i/z_A\right|\right)D_i c_i^0}{\delta}-\frac{\left|z_i\right|D_i c_i^0}{\left|z_A\right|D_A c_A^0}\cdot j_{A\lim}^{theor} \quad \text{(S27)}$$

The first term in Eq. (S27) shows what value the limiting flux density of counterion i would have if the membrane were impermeable to coions. The second term reflects the additional value of the counterion flux caused by the transfer of coions: when the coions appear in the depleted layer, they create an additional electric field that attracts counterions from the solution. This effect is called exaltation in the literature [20,21].

From Eq. (S27), it is easy to obtain an expression for the limiting current density:

$$i_{\lim}=z_1 j_1+z_2 j_2+z_A j_A=i_{\lim}^0+\left(\frac{T_{A_{\lim}}}{t_A}\right)i_{\lim} \quad \text{(S28)}$$

where $t_A=\frac{D_A z_A^2 c_A^0}{\sum_{i=1,2,A} D_i z_i^2 c_i^0}$ is the coion transport number in the bulk solution, $T_{A_{\lim}}=\frac{z_A j_{A\lim}^{theor}F}{i_{\lim}}$ is the coion effective transport number in the membrane at $i=i_{\lim}$,

$$i_{\lim}^0=\frac{\left[\left(1+\left|z_1/z_A\right|\right)D_1 z_1 c_1^0+\left(1+\left|z_2/z_A\right|\right)D_2 z_2 c_2^0\right]F}{\delta} \quad \text{(S29)}$$

is the limiting current density in the case of a membrane impermeable to coions. The term $\left(T_{A_{\lim}}/t_A\right)i_{\lim}$ in Eq. (S29) can be interpreted as the sum of the current carried by the coions $\left(T_A\cdot i_{\lim}\right)$, and the exaltation current of counterions. From Eq. (S29), we obtain:

$$i_{\lim}=\frac{i_{\lim}^0\cdot t_A}{t_A-T_A} \quad \text{(S30)}$$

The resulting Eq. (S29) generalizes the well-known Peers equation for a single electrolyte. Indeed, setting $c_2^0=0$ gives:

$$i_{\lim}=\frac{D\cdot z_1\cdot c_1^0\cdot F}{\left(T_1-t_1\right)\delta} \quad \text{(S31)}$$

where

$$D=\frac{D_1 D_A\left(z_1+\left|z_A\right|\right)}{D_1 z_1+D_A\left|z_A\right|} \quad \text{(S32)}$$

is the electrolyte diffusion coefficient.

For a mixture of two single electrolytes with a common coion (a ternary electrolyte), one can obtain equation (S33), which is similar to equation (S31) [22]:

$$i_{\lim} = \frac{D_{ter} \cdot |z_A| \cdot c_A^0 \cdot F}{(t_A - T_A)\delta} \tag{S33}$$

with D_{ter}, the effective diffusion coefficient of ternary electrolyte,

$$D_{ter} = \left[\left(1 + \left|\frac{z_1}{z_A}\right|\right) D_1 N_1 + \left(1 + \left|\frac{z_2}{z_A}\right|\right) D_2 N_2\right] \cdot t_A = \frac{\left[\left(z_1 + |z_A|\right) D_1 z_1 c_1^0 + \left(z_2 + |z_A|\right) D_2 z_2 c_2^0\right] D_A}{\sum\limits_{i=1,2,A} D_i z_i^2 c_i^0} \tag{S34}$$

$N_i = \dfrac{z_i c_i^0}{z_A c_A^0}$ is the equivalent fraction of counterion i in the bulk solution. It is easy to see that in the case, where the concentration of counterion 2 is zero, $c_2^0 = 0$, Eq. (S34) is reduced to Eq. (S32).

For a single electrolyte, the Leveque equation (Eq. (5) in the main text), allows calculating the limiting current density and diffusion layer thickness as functions of the (single) electrolyte diffusion coefficient, solution flow rate, distance between the membranes and membrane length. It can be assumed that this equation remains valid in the case of ternary electrolyte, if the value determined by Eq. (S34) is used as the electrolyte diffusion coefficient. Under this assumption, we can write

$$\delta^{Lev} = 0.68h\left(\frac{LD}{h^2 V_0}\right)^{1/3} \tag{S35}$$

Table S3 summarizes some of the characteristics of the studied electrolytes, which are used to calculate the limiting currents.

Table S3
Some characteristics of the electrolytes under study. All data refer to the temperature of 25 °C.

Electrolyte	Diffusion coefficients at infinite dilution, $D_i \times 10^5$, cm^2 s^{-1}				Transport numbers at infinite dilution, t_i		
	cation	anion		electrolyte	cation	anion	
		singly charged	doubly charged			singly charged	doubly charged
NaCl	1.334 [61]	2.032 [14]		1.61	0.396	0.604	
NaH_2PO_4		0.959 [14]	0.759 [14]	1.12	0.581	0.419	
NaH_2Cit		0.799 [23]	0.700 [14]	D_{ter}=1.02	0.576	0.263	0.161
NaHT		0.852 [24]	0.794 [14]	D_{ter}=1.10	0.528	0.229	0.243
KHT	1.957 [61]	0.852 [24]	0.794 [14]	D_{ter}=1.29	0.621	0.184	0.195

The diffusion coefficient of a proton at infinite dilution of the solution (25 °C) is 9.311×10^{5}, cm^2 s^{-1} [14].

S3.2. A one-dimensional three-layer steady-state model of weak acid species transfer in anion exchange and diffusion boundary layers (developed in Refs. [24,27])

System description

Under the influence of an electric field, concentration polarization develops on the membrane / solution interface: the solution is depleted at one side of the membrane, and enriched at the other side. The concentration changes are due to the difference in the values of ion transport numbers in the membrane and in the solution. The depleted DBL is located from $x=-\delta$ to $x=0$; the enriched DBL, from $x=d$ to $x=d+\delta$ (Figure S3), where δ and d are the thickness of the diffusion layers and the membrane, respectively. The ion-exchange membrane is located between $x=0$ and $x=d$.

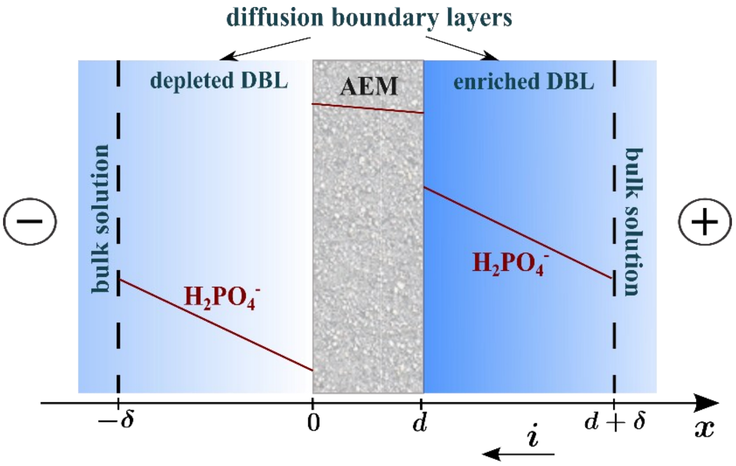

Figure S3. Schematic representation of the membrane system

The model takes into account the presence of seven different species in the membrane and the adjacent diffusion layers. We consider the presence of Na^+ and the species, which are involved in the reactions of protonation-deprotonation between different forms of phosphates and water. Table S4 shows these species and the subscripts, which are used to indicate them hereinafter.

Table S4.

Considered species

Species	Subscripts
H_3PO_4	0
$H_2PO_4^-$	1
HPO_4^{2-}	2

PO_4^{3-}	3
OH^-	4
H^+	5
Na^+	6

Chemical equilibriums in the system

The local chemical equilibrium is assumed at any point of the system:

$$K_{a,1} = \frac{c_1 c_5}{c_0}, \quad \left(H_3PO_4 \rightleftarrows H_2PO_4^- + H^+\right); \tag{S52}$$

$$K_{a,2} = \frac{c_2 c_5}{c_1}, \quad \left(H_2PO_4^- \rightleftarrows HPO_4^{2-} + H^+\right); \tag{S53}$$

$$K_{a,3} = \frac{c_3 c_5}{c_2}, \quad \left(HPO_4^{2-} \rightleftarrows PO_4^{3-} + H^+\right); \tag{S54}$$

$$K_b = c_4 c_5, \quad \left(H_2O \rightleftarrows OH^- + H^+\right). \tag{S55}$$

Here $K_{a,i}$ are the equilibrium constants of protonation-deprotonation reactions (S52) – (S54), K_b is the constant of water self-ionization.

The distribution of phosphoric acid species as a function of pH is shown in Figure S2a.

The species present in the system satisfy also the local electroneutrality condition, which is written in solution:

$$c_6 + c_5 - c_4 - 3c_3 - 2c_2 - c_1 = 0 \tag{S56a}$$

and in the membrane

$$c_6 + c_5 - c_4 - 3c_3 - 2c_2 - c_1 + c_m = 0 \tag{S56b}$$

where c_m is the concentration of the membrane fixed charges.

Eqs. (S52) – (S55) are used locally at any point of the three-layer system. These equations are used also together with the equation expressing the total concentration of phosphor (C)

$$C = c_0 + c_1 + c_2 + c_3. \tag{S57}$$

to find the concentration of each species in the bulk solution c_i^B at two external boundaries, $x = -\delta$ and $x = d + \delta$, where the Na^+ ion concentration (c_6) and the total phosphor concentration (C) are known.

Transport in the diffusion layers

The flux density of species i (j_i) is described by the Nernst-Planck equation, written in a steady state as:

$$j_i = -D_i\left(\frac{dc_i}{dx} + z_i c_i \frac{F}{RT}\frac{d\varphi}{dx}\right), \tag{S58}$$

where the gradient of electric potential $\frac{d\varphi}{dx}$ can be found by summing up Eq. (S58), written for each of the seven species and applying the local electroneutrality assumption (S56a):

$$\frac{F}{RT}\frac{d\varphi}{dx} = -\frac{\sum_i z_i j_i / D_i}{\sum_j z_j^2 c_j}. \tag{S59}$$

Here z_i and D_i are the charge and the diffusion coefficient of species i; F is the Faraday constant; R is the gas constant; T is the temperature.

The Na^+ ions are not involved in the protonation-deprotonation reactions. In the case of steady-state transport across an ideally permselective AEM considered here, the sodium ion flux density is zero at any point of the system ($j_6 = 0$). In contrast, the flux density of all other species will be a function of the coordinate, since they participate in the chemical reactions. These fluxes are calculated using the following equations:

$$i = F\sum_i z_i j_i \ ; \tag{S60}$$

$$j_T = j_0 + j_1 + j_2 + j_3 \ ; \tag{S61}$$

$$\frac{j_1}{D_1 c_1} + \frac{j_5}{D_5 c_5} = \frac{j_0}{D_0 c_0} \ ; \tag{S62}$$

$$\frac{j_2}{D_2 c_2} + \frac{j_5}{D_5 c_5} = \frac{j_1}{D_1 c_1} \ ; \tag{S63}$$

$$\frac{j_3}{D_3 c_3} + \frac{j_5}{D_5 c_5} = \frac{j_2}{D_2 c_2} \ ; \tag{S64}$$

$$\frac{j_4}{D_4 c_4} + \frac{j_5}{D_5 c_5} = 0 \ . \tag{S65}$$

where i is the electric current density; j_T is the total flux density of phosphor containing species, j_T does not depend on the coordinate in steady state due to the mass conservation law.

Eqs. (S62) – (S65) are deduced from the equilibrium equations (S52) – (S55) and the Nernst-Planck equation (S58). The same equations were used in [28] to describe the transport of 6 different species in a membrane system with a $NaHCO_3$ solution.

The effective transport number for ion *i* can be found from the obtained values of j_i:

$$T_i = \frac{j_i z_i F}{i}. \tag{S66}$$

In contrast to the conventional transport number t_i, which shows the fraction of current transported in condition $\frac{dc_i}{dx} = 0$, T_i allows the occurrence of concentration gradients. In general, T_i depends on the coordinate.

The potential difference across each diffusion layer is calculated using the Nernst-Planck equation (S58). Considering $j_6 = 0$ in steady state, Eq. (S58) can be rewritten as

$$\frac{d\varphi}{dx} = -\frac{RT}{F}\frac{d\ln c_6}{dx} \tag{S67}$$

and the potential differences in question are as follows:

$$\Delta\varphi^D = \frac{RT}{F}\ln\frac{c_6^B}{c_6^D} \; ; \tag{S68}$$

$$\Delta\varphi^E = \frac{RT}{F}\ln\frac{c_6^E}{c_6^B}, \tag{S69}$$

where c_i^D and c_i^E are the concentrations at the membrane surface in the depleted (D) and the enriched (E) diffusion layers, respectively, c_i^B is the concentration in the bulk solution.

Transport in the membrane

A membrane is modeled as a homogeneous medium containing fixed ionic groups with a molar concentration c_m.To describe the transport through the membrane and the potential difference across the membrane, a set of equations identical to those used for the diffusion layers (S58) – (S66), (S68), (S69) were applied.

To link the boundary concentrations in the membrane and the diffusion layers, the condition of electrochemical potential continuity is used. Then the following Donnan relations at the membrane interfaces with the depleted (D) and enriched (E) solutions can be found:

$$\overline{c_i^D} = c_i^D e^{-z_i F \Delta\varphi^{mD}/RT} \; ; \tag{S70}$$

$$\overline{c_i^E} = c_i^E e^{z_i F \Delta\varphi^{mE}/RT}, \tag{S71}$$

where the bar over the concentration denotes membrane phase; $\Delta\varphi^{mD}$ and $\Delta\varphi^{mE}$ are the Donnan potential drops at the respective membrane interfaces.

The system of Eqs. (S70) and (S71) written for all species and the electroneutrality equation (S52b) allow us to find the boundary concentrations of all species in the membrane and the values of both Donnan potential drops.

Model parameters

The calculations are carried out for the following parameters. The concentration of monosodium phosphate in the bulk solution is $0.02\ mol/L$, pH = 4.7. The equilibrium constants of protonation-deprotonation reactions of phosphoric acid by the first, second and third stages, as well as the water self-ionization constant are as follows [14]: $K_{a,1} = 7.25\cdot10^{-3}\ mol/L$ $(pK_{a,1} = 2.12)$; $K_{a,2} = 6.31\cdot10^{-8}\ mol/L$ $(pK_{a,2} = 7.21)$; $K_{a,3} = 4.8\cdot10^{-13}\ mol/L$ $(pK_{a,3} = 12.34)$; $K_b = 10^{-14}\ mol/L$ $(pK_b = 14)$. The diffusion coefficients of the ions and the molecules in solution are taken at infinite dilution [14]: $D_0 = 0.91\cdot10^{-9}\ m^2/s$; $D_1 = 0.958\cdot10^{-9}\ m^2/s$; $D_2 = 0.759\cdot10^{-9}\ m^2/s$; $D_3 = 0.824\cdot10^{-9}\ m^2/s$; $D_4 = 5.27\cdot10^{-9}\ m^2/s$; $D_5 = 9.3\cdot10^{-9}\ m^2/s$; $D_6 = 1.33\cdot10^{-9}\ m^2/s$.

The determination of the exact values of the diffusion coefficients in the membrane, $\overline{D_i}$, for a multicomponent system is a very complex task. For approximate evaluation of $\overline{D_i}$, note that the size of $H_2PO_4^-$, HPO_4^{2-} and PO_4^{3-} ions (being counterions for anion-exchange membranes) differ only slightly. Therefore, we can assume that the diffusion coefficients of all these ions have close values. We can approximately evaluate these diffusion coefficients as $\overline{D_i} = 2\cdot10^{-11}\ m^2/s$. The conductivity of the membrane calculated using these diffusion coefficients is equal to 0.98 *mS /cm*, which is consistent with the experimentally determined value, 1.0 *mS /cm* [29].

When we compare the calculated and experimental CVCs, we use the corrected potential drop $\Delta\varphi'$ defined using Eq. (S72).

S3.3. Details of the simulation of overlimiting mass transfer at the undulated surface of the anion exchange membrane in overlimiting current modes

The calculations were performed using the so-called "base model", which is based on the two-dimensional equations of the Nernst-Planck-Poisson-Navier-Stokes and takes into account

the electroconvective transfer of ions and fluid [30]. This model is successfully used to describe the overlimiting mass transfer in extended desalination channels of electrodialyzers. In our case, the membrane has a geometrically inhomogeneous (undulated) surface, characterized by a doubled amplitude, A, and a wave length, B (Fig.S.4). The solution between the anion-exchange membrane (supposedly impermeable to cations) and the middle of the desalination channel is considered. This version of the model is close to a real system and, we believe, allows us to characterize qualitatively the structure of the fluid vortex motion at the membrane surface, as well as to estimate the contribution of its geometric inhomogeneity to the overlimiting mass transfer. The calculation was performed for a NaCl solution with an input concentration of 0.01 mol m^{-3}. This low concentration is used to reduce the calculation time to an acceptable level [31]. Other input parameters of the calculation were taken to be equal to the parameters of the experimental system. A numerical solution was found by the finite element method using the commercially available COMSOL Multiphysics 5.5 software .

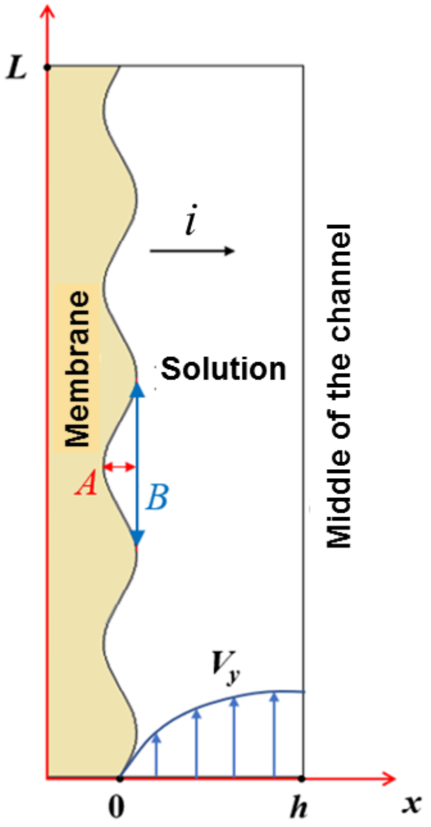

Figure S4. Schematic representation of the simulated system

S4. *Methods*

S4.1. Measurements

Figure S4 shows the scheme of the set-up and cell. The active membrane area is 2×2 cm^2; the distance between the neighboring membranes, h, is 6.6 mm; the electrolyte solution flows with a velocity of 0.4 cm s^{-1}. For measuring the potential drop over a membrane, two Luggin's capillaries were installed from the membrane left- and right-hand sides, the distance between the capillary tip and the studied membrane is 0.8 mm. The auxiliary cation-exchange (MK-40) and anion-exchange (MA-41) membranes shield the studied membrane from electrode reactions products; MK-40 and the studied membranes form a desalination compartment (14). The investigated H^+ and OH^- ions generation occurs at the surfaces of these membranes facing the desalination compartment. The difference between values of pH of the solution at the inlet and outlet of this compartment is governed by the difference between the proton and hydroxyl ions fluxes through the cation exchange membrane and the anion exchange membrane, respectively. These values are measured using combined electrodes for pH measurements (11) (Figure S5). One of them is immersed in the flow-through cell (9), the other one is in the additional tank (2). Both electrodes are connected to pH-meters (10).

The plexiglass frames that separate the membranes in the cell were equipped with special comb-like guides (Fig.S5b) to assure a laminar solution flow in intermembrane space [32].

The thickness of the swollen membrane (d_m, μm) was controlled by a high-precision digital micrometer MKC-25 0.001 with an accuracy of 1 μm. The membrane thickness was obtained by averaging the results of 10 measurements made at various points of the sample under study.

The specific electric conductivity of IEM (κ^*) was determined by a differential method using a clip cell [33,34] and an immittance meter MOTECH MT4080 (Motech Industries Inc., Taiwan) at an alternating current frequency of 1 kHz. All samples were studied in 0.02 M investigated solutions, starting from the lowest concentration.

The electric conductivity of membranes (κ^*) was determined by the equation

$$\kappa^* = \frac{d_m}{R_{m+s} - R_s} \qquad \text{(S72)}$$

where R_{m+s} is the resistance of membrane and solution; R_s is the resistance of solution; d_m is the membrane thickness in the solution of given concentration.

The current-voltage characteristics *(CVC)* were recorded at the current sweep of 0.02 mA s^{-1}. The working area of measuring Ag/AgCl electrodes immersed in the solution identical to the feed solution is 11.2 cm^2. In the case of phosphate, tartrate or citFigrate containing solution, a small amount of NaCl (~1%) was added to the solution to ensure the stable operation of the

measuring electrodes. The initial volume (before the experiment) of the feed solution in tank (1) and the hoses was 5 L. The feed solution was pumped from tank (1) through all the cell compartments; then, it returned into the same tank. The salt concentration in the feed solution circulating through tank (1) changed very little (<1%) during one experimental run due to the relatively large volume of this solution.

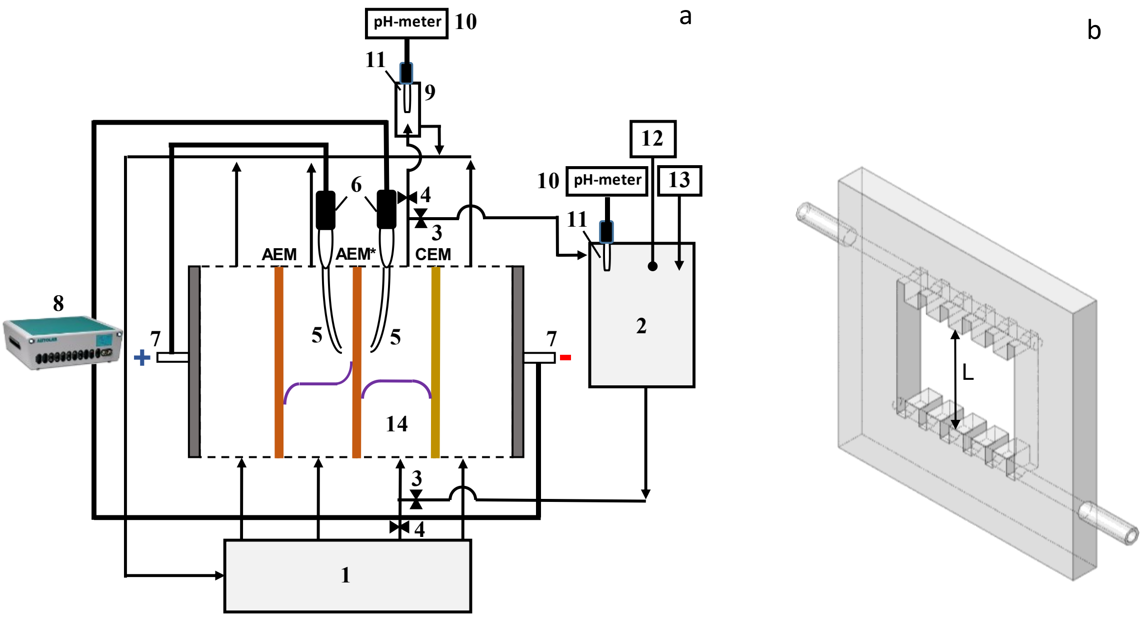

Figure S5. Schematic design of the experimental setup (a) and plexiglass frames with special comb-shaped guides that separate the membranes (b): a flow-through four-compartment electrodialysis cell containing an anion-exchange membrane under study (AEM*) and two auxiliary membranes, an anion-exchange and a cation-exchange membranes; tank with 0.02 M electrolyte solutions (1); additional tank (2) for determination of ion transport numbers; valves (3, 4); the Luggin capillaries (5); Ag/AgCl electrodes (6); platinum polarizing the working and counter electrodes (7); Autolab PGSTAT100N (8); flow-through cell with a pH combination electrode (9); pH meter pHM120 MeterLab (10) connected to computer; pH meter (10); combined electrode for pH measurements (11) connected to pH meter (10); conductivity cell (12) connected to a conductometer; titration device (13) for maintaining a constant pH in the solution circulating through tank (2); desalination compartment (14); the solid purple lines show schematic concentration profiles in two neighboring compartments separated by the membrane under study.

The potential drop over the membrane under study (AEM*) measured using Luggin's capillaries (5) is a function of the distance between the capillary tip and the membrane as well as the ohmic resistance produced by the membrane [32]. When the cell is disassembled for replacing the

membrane, these parameters change. To exclude this ambiguity, instead of the total potential drop, $\Delta\varphi$, we use the reduced potential drop, $\Delta\varphi'$, defined as follows [35]:

$$\Delta\varphi' = \Delta\varphi - iR_{ef} \tag{S73}$$

where R_{ef} (Ohm cm^2) is the effective resistance, which is found by extrapolation $i \to 0$ in the coordinates $i - d\varphi / di$, using the initial part of CVC [32].

In the case of chronopotentiograms (ChP) the reduced potential drop, Δφ', defined as follows:

$$\Delta\varphi' = \Delta\varphi - \Delta\varphi_{Ohm} \tag{S74}$$

where $\Delta\varphi_{Ohm}$ is the ohmic potential drop just after the current is switched on.

The effective transport numbers, T_i ***and partial current numbers*** i_i ***of counter ions*** were found also using the cell presented in Figure S4. The difference with the measurements of CVC is in the fact that desalination compartment (14) is fed from additional tank (2). The volume of the solution circulating through the desalination compartment and tank (2) is 0.1 L, which is essentially less than the volume of the solution circulating through tank (1), the concentration and electrode compartments. During one experimental run, in conditions where the potential difference between the Luggin capillaries, $\Delta\varphi$, is kept constant, the salt concentration in the desalination circuit decreased with time. Since the rates of generation of H^+ and OH^- ions at the CEM and AEM forming the desalination compartment are different, pH of the feed solution changed with time. Namely, it became acidic in the studied cases. In order to keep a constant pH value of the feed solution, a 0.1M solution of NaOH was added into tank (2) through microcapillary (13). The rate of decrease in the salt concentration of the solution in tank (2), *dC/dt*, is found by using the measured values of conductivity, κ, of this solution (using submersible conductometric cell (12)) and taking into account the known constant pH value. The effective transport numbers, T_i, of salt and water ions in the anion-exchange membrane under study (AEM* in Figure S4) are found by knowing the rate of concentration decrease *dC/dt*, and the rate of addition of NaOH into tank (2); the mass balance equations are applied in the calculations. Simultaneously, the partial current density of species "*i*", i_i, is determined as $i_i = i\ T_i$.

For the desalted *NaCl solution*, the material balance of the transport of counterions in the desalting channel is described by Eq. (S75), if we assume that the difference in electrolyte concentration in different parts of the installation (tube, cell, vessel) is insignificant:

$$\overline{V}\frac{dC}{dt} = -\frac{i(T_1^{CEM} - T_1^{AEM})S\,n}{z_1 F} + C_T\frac{d\overline{V_T}}{dt} \tag{S75}$$

Here T_i^{CEM}, T_i^{AEM} are the effective numbers of counterions in the cation-exchange and anion-exchange membranes that form the desalting channel; *S* is the active (polarizable) surface area of the membrane under study; *n* is the number of desalination chambers; $\overline{V}$ is the volume of the solution in the desalting channel; C_T and $\overline{V_T}$ are concentration and volume of the titrant (NaOH) added in the desalination path. The first term in the right-hand side of Eq. (S75) describes the decrease in the concentration of the electrolyte in the DC due to the transfer of counterions through the corresponding membranes; the second term of the equation describes the addition of sodium ions with the titrant.

In the case when the transfer of salt co-ions through membranes can be neglected (since 0.02 M solutions used in the study are quite diluted), it follows from Eq. (S75):

$$j_{Na^+}^{CEM} = \frac{i_{Na^+}^{CEM}}{F} = \frac{iT_{Na^+}^{CEM}}{F} \approx -\frac{\overline{V}}{S}\frac{dC}{dt} + \frac{C_T}{S}\frac{d\overline{V_T}}{dt} \tag{S76}$$

$$j_{Cl^-}^{AEM} = \frac{i_{Cl^-}^{AEM}}{F} = \frac{iT_{Cl^-}^{AEM}}{F} \approx -\frac{\overline{V}}{S}\frac{dC}{dt} \tag{S77}$$

After the effective transfer numbers of salt counterions have been determined, it is easy to find the effective transfer numbers of protons (CEM) and hydroxyl ions (AEM):

$$T_{H^+}^{CEM} = 1 - T_{Na^+}^{CEM}, \qquad T_{OH^-}^{AEM} = 1 - T_{Cl^-}^{AEM} \tag{S78}$$

In the case of desalting a *NaH_2PO_4 solution*, the partial currents of protons and sodium ions in the CEM are calculated using Eqs. (S76) and (S78).

To determine the partial currents of $H_2PO_4^-$, HPO_4^{2-}, PO_4^{3-} and OH^- ions in an AEM, the material balance equations were composed on the basis of the following assumptions.

1. The total flux of pentavalent phosphorus (P) in the AEM is equal to the sum of all fluxes of phosphorus-containing ions entering the AEM from the desalination channel. Since pH in the intermediate tank, from which the solution enters the DC, is 4.6, only $H_2PO_4^-$ ions are present in it (Figure S2a). In this case:

$$j_{H_2PO_4^-}^{s} = j_P^{AEM} = j_{H_2PO_4^-}^{AEM} + j_{HPO_4^{2-}}^{AEM} + j_{PO_4^{3-}}^{AEM} = \frac{i_{H_2PO_4^-}^{AEM}}{z_{H_2PO_4^-}F} + \frac{i_{HPO_4^{2-}}^{AEM}}{z_{HPO_4^{2-}}F} + \frac{i_{PO_4^{3-}}^{AEM}}{z_{PO_4^{3-}}F} \tag{S79}$$

where the "AEM" and "s" indices refer to the anion-exchange membrane and the solution on its side adjacent to the depleted diffusion layer (Figure 3 in the main text), respectively. The value of $j_{H_2PO_4^-}^{s}$ can be easily determined experimentally from the rate of concentration decrease in the desalting stream (where the pH value is kept constant) using an equation similar to Eq. (S77):

$$j^{s}_{H_2PO_4^-} = -\frac{\bar{V}}{S}\frac{dC_{NaH_2PO_4}}{dt} \tag{S80}$$

It follows from Figure S2a that only two types of phosphorus-containing particles can be simultaneously present in the AEM, namely $H_2PO_4^-$ and HPO_4^{2-} or HPO_4^{2-} and PO_4^{3-}. The concentration of other particles in each of the three pH ranges is very low and they can be neglected. A third pair of PO_4^{3-} and OH^- ions can coexist at pH > 13.

It should be noted that protons are co-ions and are excluded from the AEM due to the Donnan effect [36]. Therefore, the pH of the internal membrane solution is 1–2 units higher than the pH of the external solution [24]. It follows from the Donnan equation that the electrostatic exclusion of co-ions increases with dilution of the external solution [24]. The results of mathematical simulation, which take into account the values of the protonation–deprotonation constants of orthophosphoric acid particles (Table S2) and the Donnan potential of the AEM [37], show that as the current density in the membrane system increases, the solution in contact with the surface of the AEM from the depleted diffusion layer becomes more and more diluted. In this case, the concentration of H^+ ions in the near-surface layer of the membrane decreases, and the pH of this layer increases. At a relatively low current density (low concentration polarization), the pH of the near-surface AEM layer is relatively low, and this layer contains $H_2PO_4^-$ and HPO_4^{2-} ions. With an increase in the current density, the pH of the near-surface layer of the AEM increases; it is enriched first with PO_4^{3-} and then with OH^-.

In the pH range from 5 to 10 (relatively low current densities), when there are only $H_2PO_4^-$ and HPO_4^{2-} ions in the AEM, the total current density, *i*, is determined by the transfer of only these ions:

$$i^{AEM}_{H_2PO_4^-} + i^{AEM}_{HPO_4^{2-}} = i \tag{S81}$$

and, in accordance with Eq. (S80),

$$j^{s}_{H_2PO_4^-} = \frac{i^{AEM}_{H_2PO_4^-}}{F} + \frac{i^{AEM}_{HPO_4^{2-}}}{2F} \tag{S82}$$

In this case, the partial currents of singly and doubly charged phosphorus-containing ions can be found by the following equations:

$$i^{AEM}_{H_2PO_4^-} = 2Fj^{s}_{H_2PO_4^-} - i \tag{S83}$$

$$i^{AEM}_{HPO_4^{2-}} = 2\left(i - Fj^{s}_{H_2PO_4^-}\right) \tag{S84}$$

In the pH range from 10 to 13.5 (high current densities), we find in a similar way:

$$i_{HPO_4^{2-}}^{AEM} = 2\left(3Fj_{H_2PO_4^-}^{s} - i\right) \quad \text{(S85)}$$

$$i_{PO_4^{3-}}^{AEM} = 3\left(i - 2Fj_{H_2PO_4^-}^{s}\right) \quad \text{(S86)}$$

Coefficients «2» and «3» in Eqs. (S72) – (S76) correspond to electric charges $z_{HPO_4^{2-}}$ and $z_{PO_4^{3-}}$.

At higher current densities, when doubly charged ions are transformed into triply charged ones ($T_{HPO_4^{2-}} = i_{HPO_4^{2-}} / i = 0$), and the pH of the surface layer of the AEM exceeds 13.5, the current in the membrane is determined by the transfer of PO_4^{3-} and OH^- . The partial currents of these ions are found as:

$$i_{PO_4^{3-}}^{AEM} = 3Fj_{H_2PO_4^-}^{s} \quad \text{(S87)}$$

$$i_{OH^-}^{AEM} = i - i_{PO_4^{3-}}^{AEM} \quad \text{(S88)}$$

The partial flux of protons entering the depleted diffusion layer (DDL) is:

$$j_{H^+}^{s} = j_{HPO_4^{2-}}^{AEM} + 2j_{PO^{3-}}^{AEM} \quad \text{(S89)}$$

Taking into account that $i_k = j_k z_k F$, the partial current density of H^+ ions in the depleted diffusion layer at the membrane surface is:

$$i_{H^+}^{s} = \frac{i_{HPO_4^{2-}}^{AEM}}{2} + \frac{2i_{PO_4^{3-}}^{AEM}}{3} \quad \text{(S90)}$$

Note that the $i^S{}_{H+}$ values are not difficult to find if the concentration (C_T) and flow rate of the titrant (W_T), continuously added to the vessel (2) of the experimental setup are known (Figure S4). In this case, the equation for calculating $i^S{}_{H+}$ is:

$$i_{H^+}^{s} = -\frac{FW_T}{S}\frac{dC_T}{dt} \quad \text{(S91)}$$

Eq. (S91) is derived under the assumption, that all protons entering the solution from the AEM/solution interface are carried out by the fluid flow from the desalination channel. This equation is convenient to use, if the solution entering the desalting channel simultaneously contains not one, but several anions (solutions of tartaric and citric acid salts), and the calculation of the partial fluxes of these anions in the membrane is difficult.

The electrochemical impedance spectra were measured by applying the same set-up as for the CVC measurements (Fig.S5) and using the same protocol as in Ref. [38]. To record one spectrum, about 2 hours is needed. Before recording the complex impedance, the membrane was initially kept for 20 min under a given direct current (DC) density *i* and then in conditions where alternating current (AC) is imposed over the DC bias. The time necessary to attain a quasistationary state at a given frequency was found automatically by Autolab PGSTAT-100.

The recording of an EIS spectrum at a given DC density is started from the smallest frequency; the overall frequency range is from 3×10^{-3} Hz to 5×10^{5} Hz. The resulting EIS spectrum is presented as the difference between two spectra: the first one was measured with the membrane and the second one, without the membrane, using the same cell [39].

S.*4.2. Specific features of the hydraulic scheme of the setup for parallel measurement of chronopotentiograms and visualization of electroconvective vortices*

The electrodialysis cell and the hydraulic circuit of the set-up for parallel measurement of chronopotentiograms and visualization of electroconvective vortices have a number of specific features. A schematic diagram of this set-up and the cell are shown in Figure 1 of the main text of the article.

Note that a steady layered unidirectional convective flow, devoid of any fluid fluctuations, is a paramount condition when organizing an experiment to study electroconvection in flow-pass channels an electrodialysis cell. To ensure these conditions, two buffer tanks (11), which are located above the cell, are used. The tank (11) is equipped with special devices that maintain a constant solution level, providing a constant hydrostatic pressure. The solution is fed into the buffer tanks (11) by means of pumps (12) from the tanks (9), and then flows by gravity into the electrodialysis cell through the holes in the plastic pressure plates. The inner diameters of the tubes and holes through which the liquid enters the cell are 3.0 mm, the cell has an intermembrane distance of 3.2 mm. The channels of the electrodialysis cell, through which the liquid is pumped, have a length of 30 mm. It includes the length of the hydraulic stabilization section after the flow has entered the cell, which is equal to 15 mm. Special devices for input and output of solutions as well as the hydraulic stabilization sections neutralize any fluctuations in the fluid flow.

The membranes in the cell were arranged vertically. The solution flowed perpendicular to the direction of the Earth's gravitational field. The average linear velocity of the solution flow is 0.07 cm s (Reynolds number Re ≈1). This value of the Reynolds number provides a steady layered hydrodynamic flow of the solution in the cell.

The electrodes polarizing the membrane system were located in the geometric center of the experimental cell. The length of these electrodes was equal to 5.3 mm. Electroconvective vortices arised and were recorded precisely in the polarized section. The ratio of the intermembrane distance to the desalination length (the length of the polarized section) was chosen in such a way as to provide the clearest image of the recorded electroconvective vortices.

The absence of fluctuations or vortex flow of a liquid without an imposed electric field has been verified repeatedly in preliminary experiments.

Previous experiments and calculations using 2D model showed [40] that gravitational convection does not occur under the conditions of the electroconvection visualization.

S.5. Some results of numerical simulation using a stationary model (Section S3.3) of the transfer of species of weak polybasic acids in a system with an anion-exchange membrane

In the case of NaH_2PO_4 solution, the model (Section S3.3) predicts the presence of two inclined plateaus on the total CVC (Figure S6). In the region of the first plateau (about 0.05 V of the reduced potential drop), the partial current density of singly-charged ions $H_2PO_4^-$ in the membrane reaches a maximum value that is close to the limiting current calculated by the Leveque equation. The limiting current calculated by the Leveque equation is the limiting value of the partial current carried by $H_2PO_4^-$ ions in solution, where this change this current is limited by diffusion through the depleted diffusion layer. When Δφ' is close to 0.05 V, the concentration of $H_2PO_4^-$ ions at the AEM surface reaches a value, which is much lower than the concentration value in the bulk solution, hence its diffusion flux density attains a maximum [26,41]. Thus, the appearance of the first plateau II' is due to the saturation of the NaH_2PO_4 salt diffusion from the solution to the membrane surface (Figure S6b).

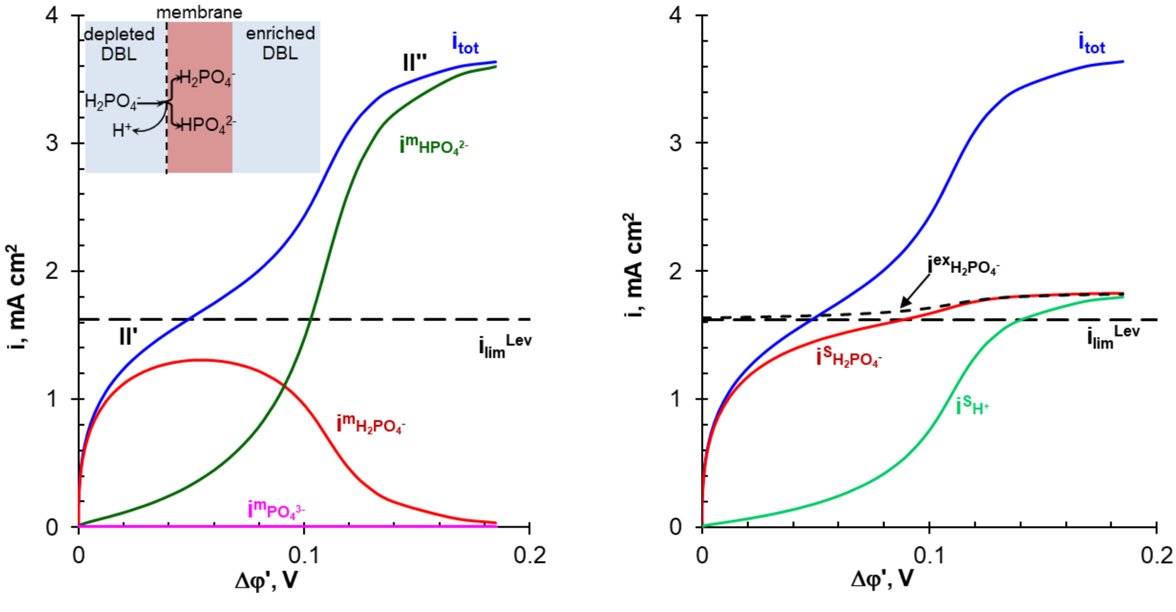

(a) (b)

Figure S6. Theoretical total current density (i_{tot}) and partial currents of $H_2PO_4^-$ ($i^{AEM}_{H_2PO_4^-}$) and HPO_4^{2-} ($i^{AEM}_{HPO_4^{2-}}$) ions in an AMX membrane (a) as well as the partial currents of $H_2PO_4^-$ ($i^{s}_{H_2PO_4^-}$) and H^+ ($i^{s}_{H^+}$) ions in the depleted solution at the membrane surface (b) as functions of the corrected potential drop. Solid lines are calculated using model [25,26]. Dashed lines show the limiting current $i^{Lev}_{\lim}$ calculated using the Leveque equation, Eq. (5) in main text,

and the exaltation current, $i^{ex}_{H_2PO_4^-}$, calculated using Eq. (S45). "II'" and "II"" show the first and second inclined plateaus, respectively.

The decrease in NaH_2PO_4 concentration in the solution at the AEM surface leads to a stronger Donnan exclusion of protons from the membrane. As a result, the pH of the AEM internal solution increases and a higher part of the singly-charged phosphate $H_2PO_4^-$ ions transform into the doubly-charged HPO_4^{2-} ions when crossing the membrane interface: $H_2PO_4^- \rightarrow HPO_4^{2-} + H^+$ (insert in Figure S6a). The protons released into solution at the depleted membrane interface are involved in the charge transfer in the depleted DBL forming partial current density i^s_{H+} (Figure S6b). When the fluxes of PO_4^{3-} and OH^- ions in the membrane are negligible, $i^s_{H^+} = i^{AEM}_{HPO_4^{2-}}$ (Figure S6a). These protons appearing in solution and the doubly-charged HPO_4^{2-} anions in the membrane cause the rise of current density above the Leveque limiting current density, i_{lim}^{Lev}. The second plateau II" (and limiting current, i_{lim2}) is observed when the membrane is completely converted into the HPO_4^{2-} form. In this state, the flux of protons, released when the $H_2PO_4^-$ anions enter the membrane and transform into HPO_4^{2-}, is saturated.

Figure S7 shows dimensionless concentration profiles of individual components of the NaH_2PO_4 solution in the anion-exchange membrane AMX and the adjacent diffusion layers in a steady state at a current density i/i_{lim}^{Lev} of 0.5, 1.1, 2.0, or 2.3.

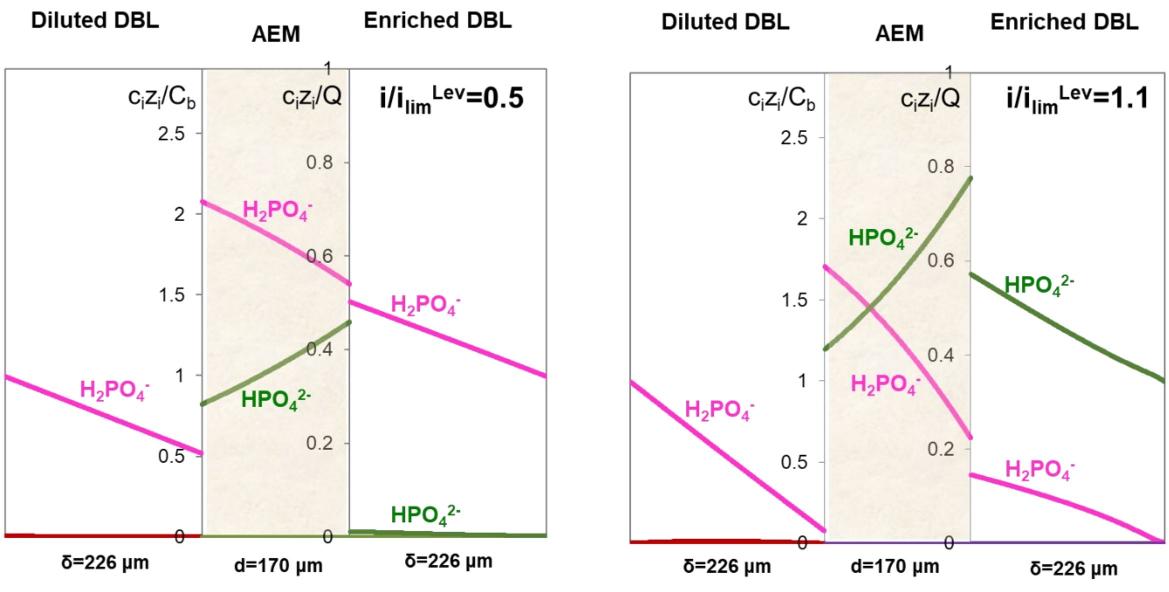

(a) (b)

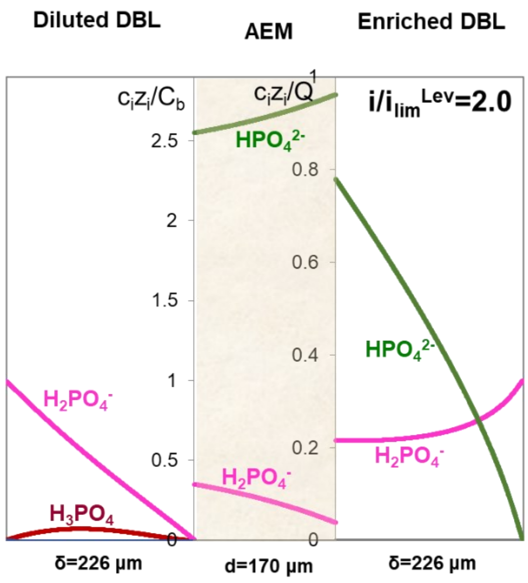

(c)

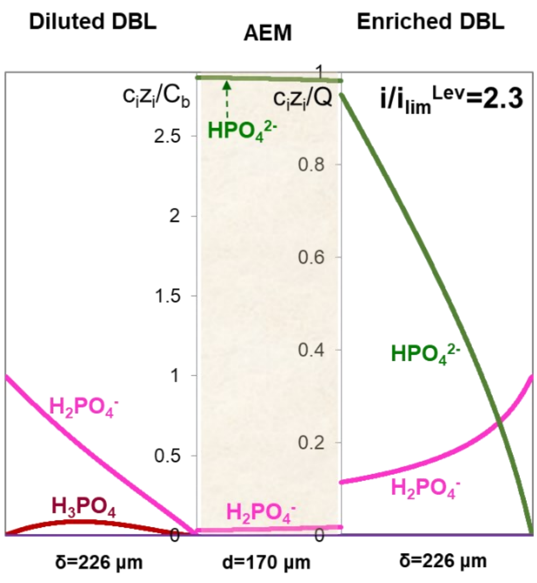

(d)

Figure S7. Dimensionless concentration profiles of individual components of the NaH_2PO_4 solution in the anion-exchange membrane AMX and the adjacent diffusion layers in a steady state at a current density i/i_{lim}^{Lev} of (a) 0.5, (b) 1.1, (c) 2.0, or (d) 2.3. Equivalent concentrations z_ic_i are used in the case of ions, and molar concentration C_i is used for neutral phosphoric acid molecules. Adapted from [42].

References

1. Y. Hori, T. Nakatani, Y.M. Morphology of Ion Exchange Membranes. *J. Electron Microsc. (Tokyo)*. **1986**, doi:10.1093/oxfordjournals.jmicro.a050571.
2. Mizutani, Y. Structure of ion exchange membranes. *J. Memb. Sci.* **1990**, *49*, 121–144, doi:10.1016/S0376-7388(00)80784-X.
3. XU, T. Ion exchange membranes: State of their development and perspective. *J. Memb. Sci.* **2005**, *263*, 1–29, doi:10.1016/j.memsci.2005.05.002.
4. Mareev, S.A.; Butylskii, D.Y.; Pismenskaya, N.D.; Larchet, C.; Dammak, L.; Nikonenko, V. V. Geometric heterogeneity of homogeneous ion-exchange Neosepta membranes. *J. Memb. Sci.* **2018**, doi:10.1016/j.memsci.2018.06.018.
5. G. Z. Nefedova, Z. G. Klimova, G.S.S. Ion-Exchange Membranes, Granulates, Powders. *Catalogue* **1977**.
6. Berezina, N.P.; Kononenko, N.A.; Dyomina, O.A.; Gnusin, N.P. Characterization of ion-exchange membrane materials: Properties vs structure. *Adv. Colloid Interface Sci.* **2008**, *139*, 3–28, doi:10.1016/j.cis.2008.01.002.

7. Pismenskaya, N.D.; Pokhidnia, E.V.; Pourcelly, G.; Nikonenko, V.V. Can the electrochemical performance of heterogeneous ion-exchange membranes be better than that of homogeneous membranes? *J. Memb. Sci.* **2018**, *566*, 54–68, doi:10.1016/j.memsci.2018.08.055.
8. Sarapulova, V. V.; Titorova, V.D.; Nikonenko, V. V.; Pismenskaya, N.D. Transport Characteristics of Homogeneous and Heterogeneous Ion-Exchange Membranes in Sodium Chloride, Calcium Chloride, and Sodium Sulfate Solutions. *Membr. Membr. Technol.* **2019**, *1*, 168–182, doi:10.1134/S2517751619030041.
9. Volodina, E.; Pismenskaya, N.; Nikonenko, V.; Larchet, C.; Pourcelly, G. Ion transfer across ion-exchange membranes with homogeneous and heterogeneous surfaces. *J. Colloid Interface Sci.* **2005**, *285*, 247–258, doi:10.1016/j.jcis.2004.11.017.
10. Gatapova N. Ts., Kader D. M., A.N. V. Research of diffusion permeability of membrane mk-40 when working in thermodynamic conditions. *Tambov Univ. Reports. Ser. Nat. Tech. Sci.* **2020**, *26*.
11. Simons, R. Electric field effects on proton transfer between ionizable groups and water in ion exchange membranes. *Electrochim. Acta* **1984**, *29*, 151–158, doi:10.1016/0013-4686(84)87040-1.
12. Simons, R. Water splitting in ion exchange membranes. *Electrochim. Acta* **1985**, *30*, 275–282, doi:10.1016/0013-4686(85)80184-5.
13. Zabolotskii, V.I.; Shel'deshov, N. V.; Gnusin, N.P. Dissociation of water molecules in systems with ion-exchange membranes. *Russ. Chem. Rev.* **1988**, *57*, 801–808, doi:10.1070/RC1988v057n08ABEH003389.
14. Lide, D.R. *CRC Handbook of Chemistry and Physics 86TH Edition 2005-2006*; 2005; ISBN 0849304792.
15. Lévêque M. A. The laws of heat transmission by convection. *Les Ann. des Mines Mem.* **1928**, *12*, 201–299.
16. Newman J., T.-A.K.E. *Electrochemical systems*; John Wiley & Sons, 2012;
17. N.P. Gnusin, V.I. Zabolotskii, V.V. Nikonenko, M.K.U. Convective-Diffusion Model of Electrodialytic Desalination. Limiting Current and Diffusion Layer. *Sov. Electrochem.* **1986**, *23*, 273–278.
18. La Cerva, M.; Gurreri, L.; Tedesco, M.; Cipollina, A.; Ciofalo, M.; Tamburini, A.; Micale, G. Determination of limiting current density and current efficiency in electrodialysis units. *Desalination* **2018**, *445*, 138–148, doi:10.1016/j.desal.2018.07.028.
19. Nikonenko, V.; Nebavsky, A.; Mareev, S.; Kovalenko, A.; Urtenov, M.; Pourcelly, G. Modelling of Ion Transport in Electromembrane Systems: Impacts of Membrane Bulk and

Surface Heterogeneity. *Appl. Sci.* **2018**, *9*, 25, doi:10.3390/app9010025.

20. Urtenov, M.A.K.; Kirillova, E. V.; Seidova, N.M.; Nikonenko, V. V. Decoupling of the Nernst-Planck and Poisson equations. Application to a membrane system at overlimiting currents. *J. Phys. Chem. B* **2007**, doi:10.1021/jp073103d.
21. Kharkats, Y.I.; Sokirko, A.V. Theory of the effect of migration current exaltation taking into account dissociation-recombination reactions. *J. Electroanal. Chem.* **1991**, *303*, 27–44, doi:10.1016/0022-0728(91)85113-4.
22. Titorova, V.D.; Mareev, S.A.; Gorobchenko, A.D.; Gil, V.V.; Nikonenko, V.V.; Sabbatovskii, K.G.; Pismenskaya, N.D. Effect of current-induced coion transfer on the shape of chronopotentiograms of cation-exchange membranes. *J. Memb. Sci.* **2021**, *624*, 119036, doi:10.1016/j.memsci.2020.119036.
23. Chandra, A.; Tadimeti, J.G.D.; Bhuvanesh, E.; Pathiwada, D.; Chattopadhyay, S. Switching selectivity of carboxylic acids and associated physico-chemical changes with pH during electrodialysis of ternary mixtures. *Sep. Purif. Technol.* **2018**, *193*, 327–344, doi:10.1016/j.seppur.2017.10.048.
24. Sarapulova, V.; Nevakshenova, E.; Pismenskaya, N.; Dammak, L.; Nikonenko, V. Unusual concentration dependence of ion-exchange membrane conductivity in ampholyte-containing solutions: Effect of ampholyte nature. *J. Memb. Sci.* **2015**, *479*, 28–38, doi:10.1016/j.memsci.2015.01.015.
25. Belashova, E.; Mikhaylin, S.; Pismenskaya, N.; Nikonenko, V.; Bazinet, L. Impact of cation-exchange membrane scaling nature on the electrochemical characteristics of membrane system. *Sep. Purif. Technol.* **2017**, *189*, 441–448, doi:10.1016/J.SEPPUR.2017.08.045.
26. Melnikova, E.D.; Pismenskaya, N.D.; Bazinet, L.; Mikhaylin, S.; Nikonenko, V. V. Effect of ampholyte nature on current-voltage characteristic of anion-exchange membrane. *Electrochim. Acta* **2018**, *285*, 185–191, doi:10.1016/j.electacta.2018.07.186.
27. Nikonenko, V.; Lebedev, K.; Manzanares, J.A.; Pourcelly, G. Modelling the transport of carbonic acid anions through anion-exchange membranes. *Electrochim. Acta* **2003**, *48*, 3639–3650, doi:10.1016/S0013-4686(03)00485-7.
28. Femmer, R.; Mani, A.; Wessling, M. Ion transport through electrolyte/polyelectrolyte multi-layers. *Sci. Rep.* **2015**, *5*, 11583, doi:10.1038/srep11583.
29. Pismenskaya, N.; Nikonenko, V.; Auclair, B.; Pourcelly, G. Transport of weak-electrolyte anions through anion exchange membranes. *J. Memb. Sci.* **2001**, *189*, 129–140, doi:10.1016/S0376-7388(01)00405-7.
30. Urtenov, M.K.; Uzdenova, A.M.; Kovalenko, A.V.; Nikonenko, V.V.; Pismenskaya,

N.D.; Vasil'eva, V.I.; Sistat, P.; Pourcelly, G. Basic mathematical model of overlimiting transfer enhanced by electroconvection in flow-through electrodialysis membrane cells. *J. Memb. Sci.* **2013**, *447*, 190–202, doi:10.1016/j.memsci.2013.07.033.
31. Druzgalski, C.L.; Andersen, M.B.; Mani, A. Direct numerical simulation of electroconvective instability and hydrodynamic chaos near an ion-selective surface. *Phys. Fluids* **2013**, *25*, 110804, doi:10.1063/1.4818995.
32. Belova, E.I.; Lopatkova, G.Y.; Pismenskaya, N.D.; Nikonenko, V. V.; Larchet, C.; Pourcelly, G. Effect of anion-exchange membrane surface properties on mechanisms of overlimiting mass transfer. *J. Phys. Chem. B* **2006**, *110*, 13458–69, doi:10.1021/jp062433f.
33. Lteif, R.; Dammak, L.; Larchet, C.; Auclair, B. Conductivité électrique membranaire: Étude de l'effet de la concentration, de la nature de l'electrolyte et de la structure membranaire. *Eur. Polym. J.* **1999**, *35*, 1187–1195, doi:10.1016/S0014-3057(98)00213-4.
34. Karpenko, L.V.; Demina, O.A.; Dvorkina, G.A.; Parshikov, S.B.; Larchet, C.; Auclair, B.; Berezina, N.P. Comparative study of methods used for the determination of electroconductivity of ion-exchange membranes. *Russ. J. Electrochem.* **2001**, *37*, 287–293, doi:10.1023/A:1009081431563.
35. Ślęzak, A.; Bryll, A.; Grzegorczyn, S. A Numerical Study of the Hydrodynamic Stable Concentration Boundary Layers in a Membrane System Under Microgravitational Conditions. *J. Biol. Phys.* **2007**, *32*, 553–562, doi:10.1007/s10867-007-9037-0.
36. Helfferich, F.G. Ion Exchange. *McGraw-Hill, New York,* **1962**.
37. Belashova, E.D.; Pismenskaya, N.D.; Nikonenko, V.V.; Sistat, P.; Pourcelly, G. Current-voltage characteristic of anion-exchange membrane in monosodium phosphate solution. Modelling and experiment. *J. Memb. Sci.* **2017**, *542*, 177–185, doi:10.1016/j.memsci.2017.08.002.
38. Rybalkina, O.; Tsygurina, K.; Melnikova, E.; Mareev, S.; Moroz, I.; Nikonenko, V.; Pismenskaya, N. Partial fluxes of phosphoric acid anions through anion-exchange membranes in the course of NaH2PO4 solution electrodialysis. *Int. J. Mol. Sci.* **2019**, doi:10.3390/ijms20143593.
39. Sistat, P.; Kozmai, A.; Pismenskaya, N.; Larchet, C.; Pourcelly, G.; Nikonenko, V. Low-frequency impedance of an ion-exchange membrane system. *Electrochim. Acta* **2008**, *53*, 6380–6390, doi:10.1016/j.electacta.2008.04.041.
40. Zabolotsky, V.I.; Nikonenko, V. V.; Pismenskaya, N.D. On the role of gravitational convection in the transfer enhancement of salt ions in the course of dilute solution electrodialysis. *J. Memb. Sci.* **1996**, *119*, 171–181, doi:10.1016/0376-7388(96)00121-4.

41. Belashova, E.D.; Melnik, N.A.; Pismenskaya, N.D.; Shevtsova, K.A.; Nebavsky, A. V.; Lebedev, K.A.; Nikonenko, V. V. Overlimiting mass transfer through cation-exchange membranes modified by Nafion film and carbon nanotubes. *Electrochim. Acta* **2012**, doi:10.1016/j.electacta.2011.10.077.
42. Belashova, E.D.; Kharchenko, O.A.; Sarapulova, V. V.; Nikonenko, V. V.; Pismenskaya, N.D. Effect of Protolysis Reactions on the Shape of Chronopotentiograms of a Homogeneous Anion-Exchange Membrane in NaH2PO4 Solution. *Pet. Chem.* **2017**, *57*, 1207–1218, doi:10.1134/S0965544117130035.
